# Histone Deacetylase Inhibitors Antagonize Distinct Pathways to Suppress Tumorigenesis of Embryonal Rhabdomyosarcoma

**DOI:** 10.1371/journal.pone.0144320

**Published:** 2015-12-04

**Authors:** Terra Vleeshouwer-Neumann, Michael Phelps, Theo K. Bammler, James W. MacDonald, Isaac Jenkins, Eleanor Y. Chen

**Affiliations:** 1 Department of Pathology, University of Washington, Seattle, Washington, United States of America; 2 Department of Environmental and Occupational Health Sciences, University of Washington, Seattle, Washington, United States of America; 3 Clinical Research Division, Fred Hutchinson Cancer Research Center, Seattle, Washington, United States of America; University of Navarra, SPAIN

## Abstract

Embryonal rhabdomyosarcoma (ERMS) is the most common soft tissue cancer in children. The prognosis of patients with relapsed or metastatic disease remains poor. ERMS genomes show few recurrent mutations, suggesting that other molecular mechanisms such as epigenetic regulation might play a major role in driving ERMS tumor biology. In this study, we have demonstrated the diverse roles of histone deacetylases (HDACs) in the pathogenesis of ERMS by characterizing effects of HDAC inhibitors, trichostatin A (TSA) and suberoylanilide hydroxamic acid (SAHA; also known as vorinostat) *in vitro* and *in vivo*. TSA and SAHA suppress ERMS tumor growth and progression by inducing myogenic differentiation as well as reducing the self-renewal and migratory capacity of ERMS cells. Differential expression profiling and pathway analysis revealed downregulation of key oncogenic pathways upon HDAC inhibitor treatment. By gain-of-function, loss-of-function, and chromatin immunoprecipitation (ChIP) studies, we show that Notch1- and EphrinB1-mediated pathways are regulated by HDACs to inhibit differentiation and enhance migratory capacity of ERMS cells, respectively. Our study demonstrates that aberrant HDAC activity plays a major role in ERMS pathogenesis. Druggable targets in the molecular pathways affected by HDAC inhibitors represent novel therapeutic options for ERMS patients.

## Introduction

Rhabdomyosarcoma (RMS) is the most common soft tissue malignancy in the pediatric population and pathologically recapitulates many of the phenotypic and biological features of embryonic skeletal muscle. Based on histologic characterization, RMS falls into two major groups in children: embryonal (ERMS) and alveolar (ARMS). Embryonal rhabdomyosarcoma (ERMS) is the most common subtype, accounting for ~60% of childhood cases. In contrast to ARMS, which is genetically characterized by fusion between *PAX3* or *PAX7* and *FOXO1* genes in the majority of cases, ERMS is characterized by complex genetic changes, involving various chromosomal gains and losses [1–3]. However, *RAS* mutations are present in at least 25% of ERMS tumors [4–7]. The prognosis for patients with relapsed or metastatic ERMS is dismal, with at least 50% of patients succumbing to the disease, underscoring the need for more effective treatment in these cases. A recent comprehensive genomic study by Shern et al. demonstrates relatively low mutational frequency in rhabdomyosarcoma, with 33 recurrently mutated genes identified, with a higher number of oncogenic mutations found in ERMS compared to ARMS [7]. The findings suggest that other molecular mechanisms, such as epigenetic regulation of driver genes, might contribute to RMS tumorigenesis. Interestingly, the same study shows that about 7.4% of fusion-negative RMS harbor mutations in BCOR, a transcriptional repressor that has been shown to interact with class I and II histone deacetylases, implicating the role of histone deacetylases in the pathogenesis of RMS.

ERMS is pathologically characterized by arrested myogenic differentiation and uncontrolled proliferation. We have previously completed a large-scale chemical genetic screen (testing ~40,000 compounds) to identify drugs that induce terminal myogenic differentiation of human ERMS [8]. One lead compound identified by our chemical screen, trichostatin A (TSA), a pan-histone deacetylase (HDAC) inhibitor, suppressed tumor growth as well as induced myogenic differentiation of tumor cells *in vitro*. Histone deacetylases are one of the many classes of enzyme that participate in the epigenetic or post-translational modifications of the genome. There has been increasing interest in the therapeutic use of HDAC inhibitors in some cancers due to their potent effect on proliferating malignant cells in comparison to non-neoplastic counterparts. A large number of HDAC inhibitors are in various phases of clinical trials [9, 10]. Hematologic malignancies in particular have responded well to treatment by HDAC inhibitors, such that vorinostat and romidepsin have been approved by the Federal Drug Administration (FDA) for treatment of refractory and advanced cutaneous T-cell lymphoma (CTCL) [11, 12]. There are only a few reports of tumor suppressive effects secondary to HDAC inhibition on several rhabdomyosarcoma cell lines *in vitro* [13, 14]. A study in a mouse model of ARMS demonstrates a differential response to entinostat treatment depending on the myogenic lineage of origin of tumor cells [15]. The role of histone deacetylase activity in ERMS tumorigenesis and the fundamental mechanisms by which HDAC inhibitors exert their anti-tumor effects are largely unknown.

In this study, we have revealed diverse roles of HDACs in ERMS tumorigenesis by characterizing the effects of two pan-HDAC inhibitors, trichostatin A (TSA) and suberoylanilide hydroxamic acid (SAHA; also known as vorinostat) *in vitro* and *in vivo* using human RMS cell lines and a zebrafish model of ERMS. In addition to suppressing tumor growth by inducing tumor cell differentiation and reducing self-renewal capacity, TSA and SAHA also inhibit the migratory capacity of ERMS tumor cells. By expression profiling and functional analysis of differentially expressed target genes, we show that HDAC inhibitors exert their anti-tumor effects by antagonizing distinct molecular pathways. Using a chemical genetic approach, our study demonstrates that aberrant HDAC activity is a major driver of ERMS pathogenesis. Key pathways targeted by HDAC inhibitors represent potential options for novel targeted therapies in ERMS.

## Results

### HDAC inhibitors suppress ERMS growth by inducing myogenic differentiation and reduce self-renewal and migratory capacity *in vitro*

**Fig 1:**
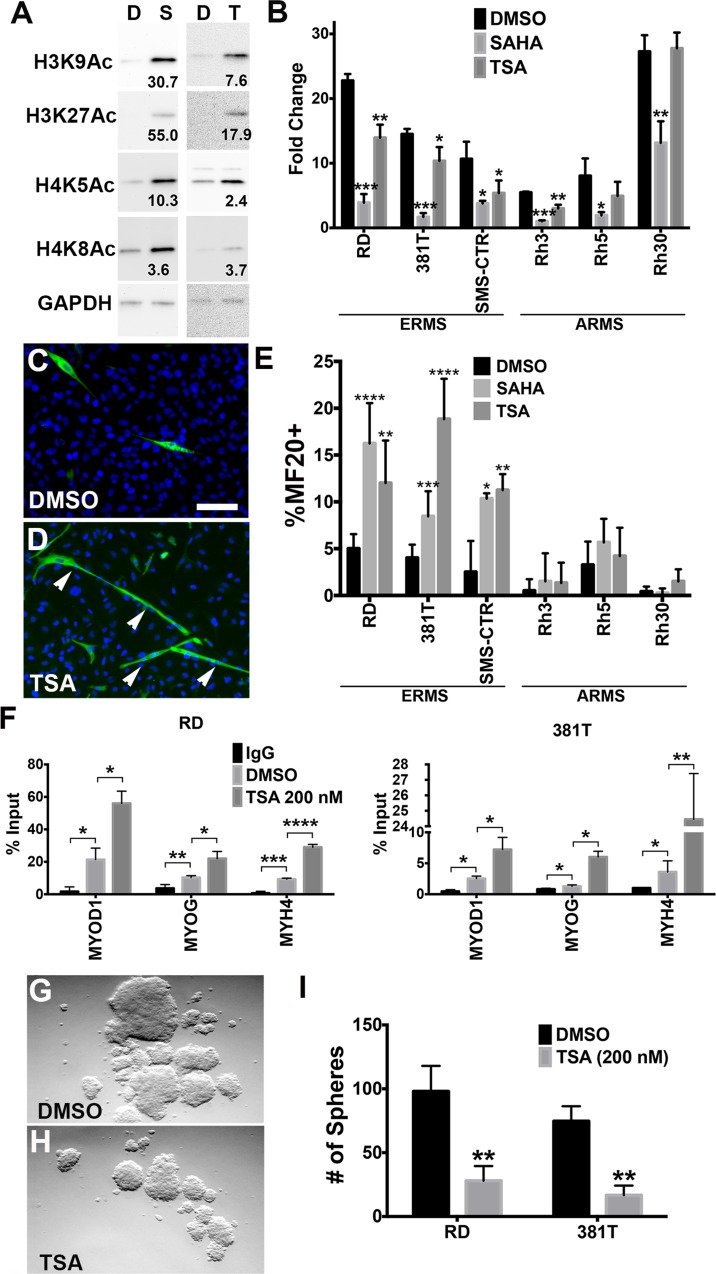
Trichostatin A (TSA) reduced growth and altered differentiation and self-renewal capacity of ERMS cell lines. (A) Western blots demonstrating hyperacetylation of histones using antibodies against acetyl-histone H3 (Lys9), acetyl-histone H3 (Lys27), acetyl-histone H4 (Lys5), and acetyl-histone H4 (Lys8). D: DMSO; S: vorinostat (SAHA); T: TSA. Each band intensity was normalized to GAPDH loading control. Relative fold increase to DMSO (vehicle) treatment is shown. (B) Analysis of cell viability by cell counts. Cell counts were performed on cells treated with DMSO, 200 nM TSA or 1 μM SAHA at day 0 and day 5. Fold change in cell counts was normalized to day 0. (C-D) Representative images of immunofluorescence (IF) against MF20 performed on RD cells treated with DMSO (C) or 200 nM TSA (D) for 3 days in 2% horse serum in DMEM. Green: MF20-positive cells. Blue: DAPI. Scale bar: 20 μm. (E) Summary of IF against MF20 in ERMS (RD, 381T and SMS-CTR) and ARMS (Rh3, Rh5 and Rh30) cell lines treated with DMSO, 200 nM TSA or 1 μM SAHA. (F) Chromatin immunoprecipitation (ChIP) assays showing differential binding of acetyl-histone H3 (Lys9) at myogenic promoters. Fold enrichment binding of *MYOD1*, *MYOG*, and *MYH4* promoter regions were determined by quantitative PCR, normalizing amplification levels to input DNA of each sample. Rabbit IgG was used as a negative control for chromatin immunoprecipitation. (G-H) Representative bright-field images from a sphere assay on RD cells treated with DMSO (G) and 200 nM TSA (H). (I) Summary of sphere assays in RD and 381T cells. Each error bar in panels (B), (E), (F) and (I) indicates standard deviation of 3 technical replicates. * indicates p < 0.05. ** indicates p < 0.01. *** indicates p < 0.001.

The pan-HDAC inhibitor, trichostatin A (TSA), was previously identified in a high-throughput screen as a lead compound with the capacity to induce myogenic differentiation of human ERMS cells [8]. To test the effects of HDAC inhibitors on tumor growth and differentiation *in vitro*, two pan-HDAC inhibitors, TSA and suberoylanilide hydroxamic acid (SAHA), were tested in a panel of ERMS cell lines (RD, 381T and SMS-CTR) and ARMS cell lines (Rh3, Rh5 and Rh30). TSA or SAHA treatment resulted in hyperacetylation of histones including acetyl-histone H3 (Lys9), acetyl-histone H3 (Lys27), acetyl-histone H4 (Lys5), and acetyl-histone H4 (Lys8) (results for RD cells shown in Fig 1A), indicating that TSA and SAHA can alter the histone acetylation landscape in RMS cells. By quantitative analysis of cell numbers and an ATP-based cell viability assay, TSA and SAHA inhibited tumor growth in all ERMS cell lines tested (Fig 1B; S1A and S1B Fig). By contrast, ARMS cell lines exhibited a variable response to TSA and SAHA (Fig 1B). Within the ARMS cell lines, SAHA inhibited tumor growth of all three lines, while TSA inhibited tumor growth only in the Rh3 cell line at doses that showed effects in all ERMS cell lines tested. Overall, TSA and SAHA appeared to exert a more potent effect on tumor growth in ERMS cell lines in comparison to ARMS cell lines. Upon cell cycle analysis, ERMS cells treated with TSA or SAHA showed a reduction in S-phase and prolonged G2/M phase (S1C and S1D Fig). TSA or SAHA treatment did not result in any significant apoptosis at doses (200 nM of TSA and 1 μM of SAHA) that induced abnormal cell cycle progression (S1E and S1F Fig). Together, these results indicate that pan-HDAC inhibitors reduce ERMS tumor growth by altering cell cycle progression.

To assess the effect of TSA and SAHA on differentiation of RMS cells, immunofluorescence against myosin heavy chain (MF20), a late myogenic differentiation marker, was performed on the same panel of RMS cell lines (RD, 381T, SMS-CTR, Rh3, Rh5 and Rh30). In contrast to the ARMS cell lines (Rh3, Rh5 and Rh30), ERMS cell lines (RD, 381T and SMS-CTR) treated with TSA or SAHA showed at least 2-fold increase in the percentage of MF20-positive cells (Fig 1C–1E), suggesting that the effect of TSA and SAHA on myogenic differentiation is specific to the ERMS subtype. Chromatin immunoprecipitation (ChIP) analysis revealed 2 to 4-fold differential enrichment of acetyl-histone H3 (Lys9) binding on promoters of a number of myogenic genes such as MYOD1, MYOG and MYH4 in ERMS cells treated with TSA compared to the cells treated with vehicle DMSO (Fig 1F), suggesting that these genes are epigenetically regulated by HDAC(s) to drive myogenic differentiation in ERMS. We then performed quantitative RT-PCR to assess the mRNA expression of myogenic genes in ERMS cells treated with TSA or SAHA. At 24 hrs post-TSA treatment, there was variable upregulation of MYOD1 and MYOG and significant upregulation of myosin heavy chain (MHC) in ERMS cells (S2A–S2C Fig). By contrast, expression of MYOD1 and MYOG was already downregulated in ERMS cells 24 hours post-SAHA treatment, and there was significant upregulation of MHC in ERMS cells (S2D–S2F Fig). As muscle cells undergo differentiation, MYOD1 and MYOG expression levels are downregulated while late-differentiation myogenic genes, such as myosin heavy chains, are upregulated. Overall, TSA and SAHA induced distinct kinetic changes in the expression levels of myogenic factors in ERMS cells, and the expression profiles reflect a late-differentiation myogenic program by 24 hours post-treatment.

The sphere assay has previously been demonstrated as a powerful surrogate assay to assess self-renewal potential in a variety of tumor types including ERMS [8, 16]. Here we showed that SAHA or TSA treatment resulted in at least 2-fold reduction in sphere formation of human ERMS cells (Fig 1G–1I; S2G and S2H Fig), and this effect was further enhanced upon serial replating (S3I and S3J Fig), suggesting that inhibition of HDAC function can reduce self-renewal potential of human ERMS cells. Together, the results of our *in vitro* studies implicate the essential role of HDACs in modulating the differentiation and self-renewal status of ERMS during tumor progression. As the self-renewal capacity of tumor cells is an indicator of their relapse potential, HDAC activity may serve as a potential biomarker for poor prognosis.

**Fig 2:**
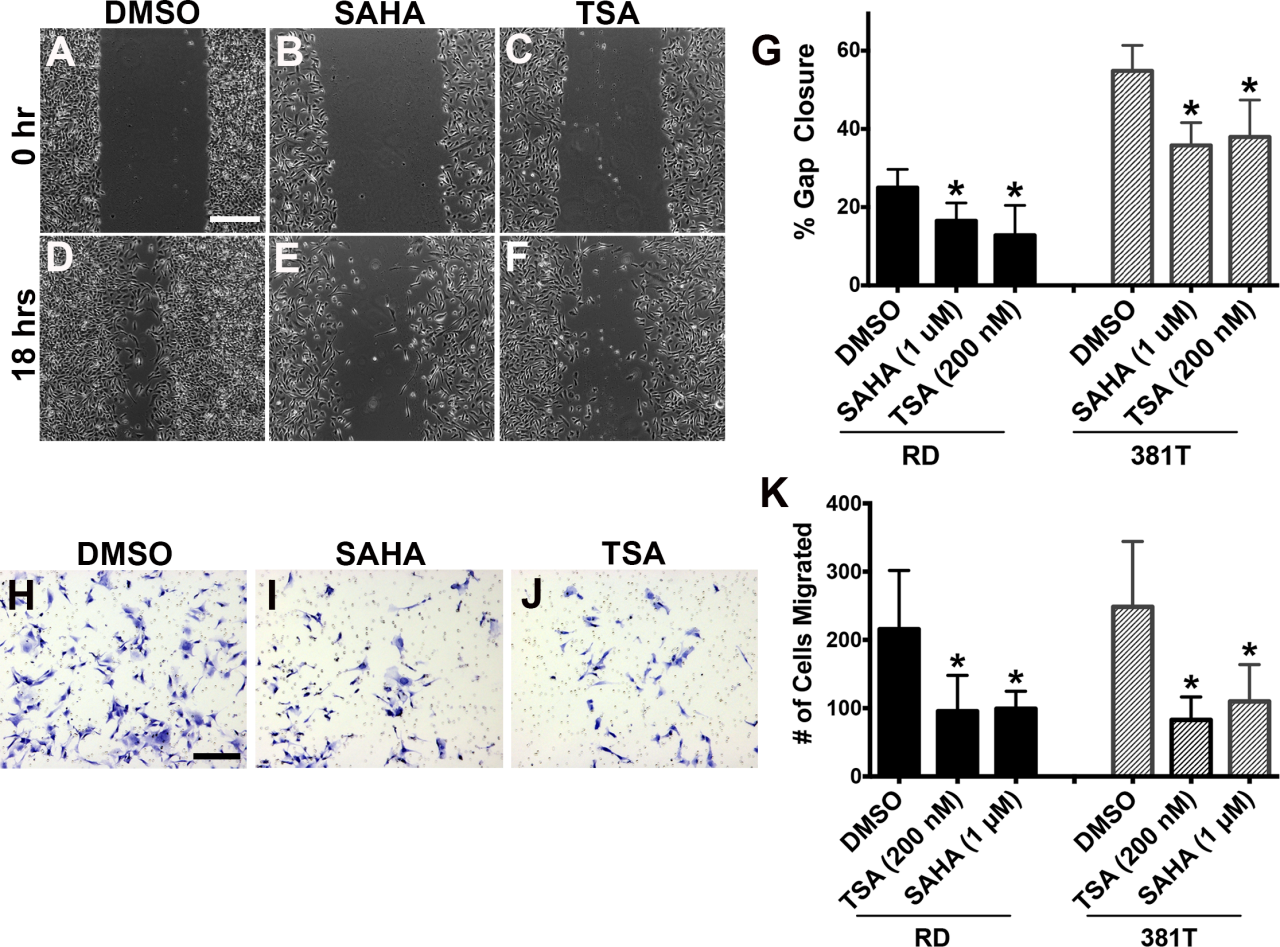
TSA and SAHA reduced migratory capacity of ERMS cells. (A-F) Scratch assays on RD cells treated with DMSO, 1 μM SAHA or 200 nM TSA. (A-C) Representative images at time of scratch (0 hr). (D-F) Representative images at 18 hours post-scratch. Scale bar = 500 μm. (G) Summary of scratch assays in RD cells, indicating % wound closure for each treatment. (H-J) Representative images post-22 hour migration from transwell assays on RD cells treated with DMSO, 1 μM SAHA or 200 nM TSA. Scale bar = 50 μm. (K) Summary of transwell assays. Each error bar in panels (G) and (K) indicates standard deviation from technical triplicates. * indicates p < 0.05.

Pan-HDAC inhibitors have been shown to elicit a variety of anti-tumor effects in other cancer types, including a reduction in migratory and invasive behaviors [9, 10]. Wound closure scratch and transwell assays were used to assess the effect of TSA and SAHA on the migratory capacity of human ERMS cells. In contrast to DMSO treatment, ERMS cells treated with TSA or SAHA showed a significant reduction in the percentage of gap closure in scratch assays (Fig 2A–2G) as well as decreased numbers of migrating cells in the transwell assays (Fig 2H–2K). The reduction in the amount of migratory ERMS cells upon TSA or SAHA treatment was not due to increased apoptosis (S1E and S1F Fig). Taken together, pan-HDAC inhibitor treatment reduced the migratory capacity of ERMS cells, implicating the role of HDACs in modulating the invasive and metastatic behavior of ERMS cells.

### Pan-HDAC inhibitors induce tumor cell differentiation and reduce self-renewal capacity of ERMS *in vivo*

**Fig 3:**
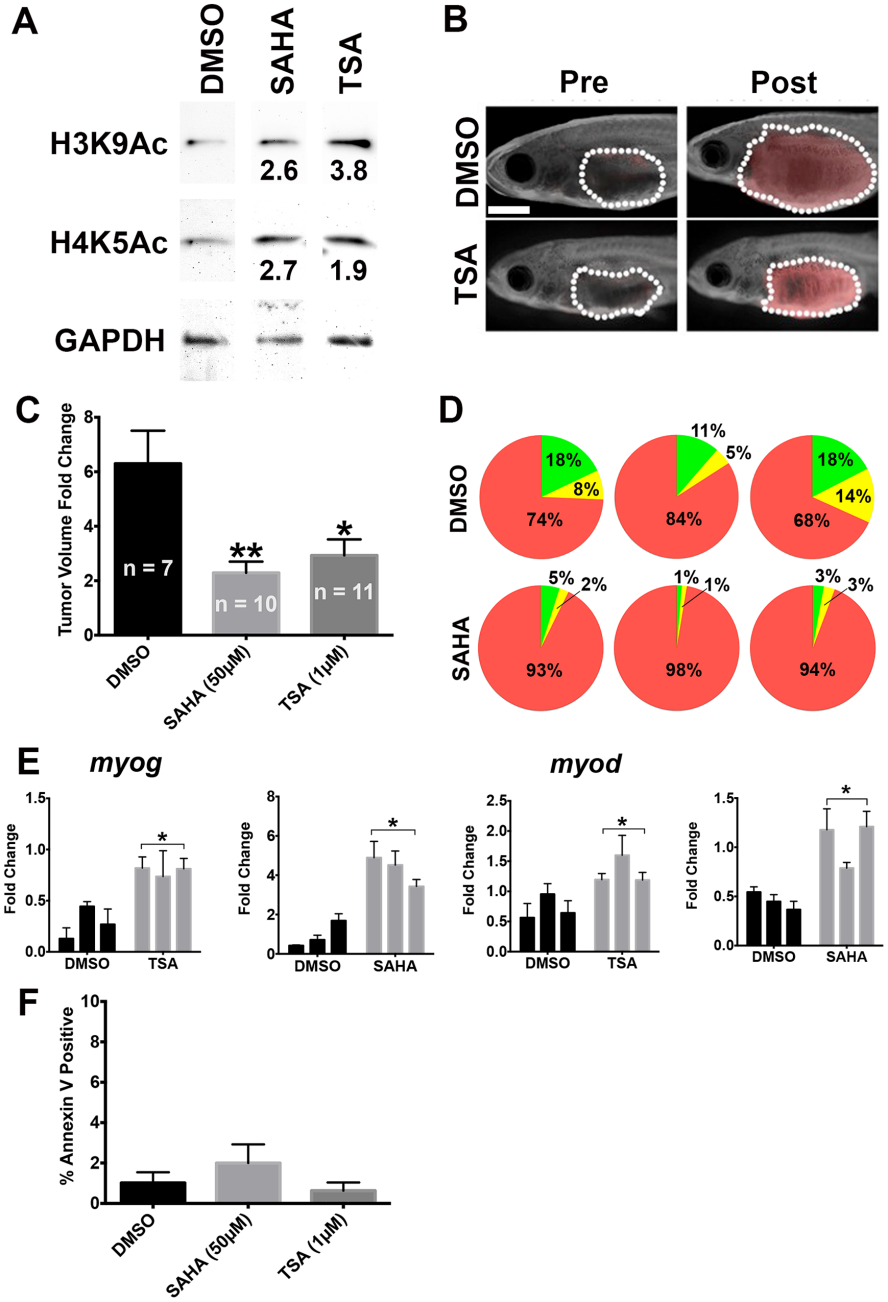
TSA and SAHA reduced tumor growth and induced myogenic differentiation *in vivo*. (A) Western blots demonstrating hyperacetylation of histone H3 (Lys9) and histone H4 (Lys5) in zebrafish ERMS treated with 1 μM TSA or 50 μM SAHA. GAPDH was used as loading control. The values shown represent fold change in band intensity from TSA or SAHA treatment relative to DMSO after normalizing to loading control. (B) Representative pre- and post-treatment images of zebrafish ERMS treated with DMSO (vehicle) or 1 μM TSA. Dotted line outlines the tumor in each fish. Scale bar = 2 mm. (C) Summary of tumor volume change of zebrafish Tg(*myf5*:GFP; *mylz2*:mCherry) ERMS treated with DMSO, 50 μM SAHA or 1 μM TSA. Overlaid images of bright field and red fluorescent channel are shown. Error bar indicates standard error of means. n = number of animals treated in each cohort. (D) Summary of quantitative Fluorescence Activated Cell Sorting analysis on ERMS treated with DMSO or 10 μM SAHA. Each pie chart shows relative proportion of each tumor cell subpopulation in an individual treated tumor. Green: *myf5*:GFP^+^/*mylz2*:mCherry^−^cells; yellow: *myf5*:GFP^+^/*mylz2*:mCherry^+^ cells; red: *mylz2*:mCherry^+^/*myf5*:GFP^−^cells. (E) Quantitative RT-PCR analysis of *myog* and *myod* mRNA expression in ERMS treated with DMSO, 1 μM TSA or 50 μM SAHA. Each bar demonstrates an individual tumor. Each error bar indicates standard deviation of technical triplicates. (F) Annexin V analysis of ERMS tumors treated with DMSO, 50 μM SAHA, or 1μM TSA. 6 animals were analyzed per group. Each error bar indicates standard deviation. * indicates p < 0.05. ** indicates p < 0.01.

As treatment of human ERMS with TSA and SAHA resulted in reduced tumor cell growth, increased tumor cell differentiation and reduced self-renewal capacity *in vitro*, we then assessed whether these effects could be recapitulated *in vivo* using a zebrafish transgenic ERMS model [17, 18]. In this transgenic model, primary ERMS was induced in the skeletal muscle by overexpression of the constitutively active human KRASG12D driven by the *rag2* promoter in wild-type zebrafish [18]. Zebrafish ERMS tumor cell subpopulations can be labeled with fluorescent reporters based on their differentiation status to allow for cellular and molecular characterization of tumor cell subpopulations [17]. Specifically, *myf5*:GFP labels tumor-propagating cells and early myoblasts, and *mylz2*:mCherry labels late-differentiating myoblasts. The *myf5*:GFP^+^/*mylz2*:mCherry^−^cell population enriches for the tumor propagating cell subpopulation and is the only cell population in this ERMS model to have self-renewing capacity. We first showed by Western blot analysis that TSA or SAHA treatment resulted in increased acetylation of histones in zebrafish ERMS tumors, indicating an altered histone acetylation status in ERMS *in vivo* (Fig 3A). ERMS tumor-bearing fish treated with TSA or SAHA showed significantly reduced tumor growth compared to a DMSO-treated cohort (n = 7 for DMSO, n = 10 for SAHA, n = 11 for TSA; Fig 3B and 3C). By quantitative Fluorescent Activated Cell Sorting (FACS) analysis, tumor cells treated with TSA or SAHA also showed alterations in differentiation, with depletion of *myf5*:GFP^+^/*mylz2*:mCherry^−^tumor cell subpopulation and expansion of the late-differentiating *mylz2*:mCherry^+^/*myf5*:GFP^−^tumor cell subpopulation (results for SAHA treatment shown in Fig 3D). ERMS cells isolated from tumor-bearing fish treated with TSA or SAHA also showed concomitant upregulation of the myogenic regulatory factors *myogenin* and *myod* (Fig 3E), indicating an induction of the myogenic differentiation program. TSA or SAHA treatment did not result in an increase in the apoptosis of ERMS cells (Fig 3F), indicating that apoptosis does not contribute to reduced tumor growth or self-renewal frequency from HDAC inhibitor treatment.

As HDAC inhibitor treatment resulted in depletion of the *myf5*:GFP^+^/*mylz2*:mCherry^−^tumor cell subpopulation, which contains the tumor propagating cells with the capacity for self-renewal [17, 18], we next assessed the effect of HDAC inhibitors on the self-renewal capacity of ERMS *in vivo* by limiting dilution experiments. Either unsorted bulk tumor cells or *myf5*:GFP^+^/*mylz2*:mCherry^−^sorted cells were transplanted at limiting dilutions into syngeneic juvenile fish hosts and subsequently treated with DMSO (vehicle) or HDAC inhibitor (TSA or SAHA) for 5 days prior to the monitoring period for tumor engraftment. Compared to the DMSO-treated cohort, TSA or SAHA-treated tumor-bearing fish showed approximately 3 to 10-fold reduction in the self-renewal frequency (p < 0.05, limiting dilution experiments using four independent primary tumors are shown in Tables 1–3). Together, our results from characterizing the effects of HDAC inhibitors in human ERMS cell lines and in the zebrafish ERMS model suggest an important role of HDACs in ERMS tumorigenesis by regulation of the balance between differentiation and self-renewal of tumor cells.

**Table 1:** Summary of limiting dilution experiment of myf5-GFP+ sorted transplants treated with SAHA.

Cell No.	DMSO	SAHA
10^3	NA	NA
10^2	3 of 8	1 of 6
10	3 of 10	0 of 7
**TPC frequency**	1 in 119	1 in 619*
**95% CI**	50–284	88–4367

TPC: Tumor propagating frequency.

* indicates p < 0.05.

**Table 2:** Summary of limiting dilution experiments of unsorted bulk tumor treated with SAHA.

Cell No.	DMSO	SAHA
5^3	7 of 8	3 of 6
5^2	4 of 4	6 of 6
50	1 of 4	0 of 5
**TPC frequency**	1 in 130	1 in 2420*
**95% CI**	38–443	1002–5849

TPC: Tumor propagating frequency.

* indicates p < 0.05.

**Table 3:** Summary of limiting dilution experiments of unsorted bulk tumor treated with TSA.

Cell No.	Experiment #1		Experiment #2	
	DMSO	TSA	DMSO	TSA
10^4	NA	NA	9 of 9	7 of 8
10^3	7 of 9	4 of 9	3 of 6	4 of 4
10^2	6 of 10	3 of 7	4 of 7	1 of 5
**TPC frequency**	1 in 366	1 in 1033*	1 in 662	1 in 2283*
**95% CI**	175–766	458–2329	272–1609	808–6454

TPC: Tumor propagating frequency.

* indicates p < 0.05.

### NOTCH1 loss-of-function phenocopies HDAC inhibitor-induced effects on differentiation of ERMS

To identify candidate genes that are differentially regulated in HDAC inhibitor-treated ERMS, a gene expression profiling study using the Affymetrix Human Gene 2.0 microarray platform was performed on RD and 381T cells treated with TSA or DMSO, and Ingenuity Pathway Analysis was performed on differentially expressed genes. Instead of global change in gene expression, TSA-treatment resulted in a restricted set of differentially-expressed genes. We focused on validating candidate genes with potential signaling or oncogenic function (Table 4). Among the top upregulated gene candidates, we have validated MPP4 (annotated function of extracellular matrix remodeling function), CPA4 (extracellular peptide processing), SEMA3C (migration and morphogenesis) as well as DNER, PTPRN and TNFRSF12A, genes involved in cytokine-mediated signaling activity. Among top genes/pathways downregulated upon TSA treatment, FGFR1, SMO (receptor for the Hedgehog pathway) and Notch1 have been previously been implicated in promoting tumorigenesis of ERMS [3, 7, 19–21].

**Table 4:** Validation of top downregulated and upregulated genes in TSA-treated ERMS cells.

**Top downregulated genes**	**Fold Change**	**STDEV**
**FGFR1**	0.48	0.17
**SMO**	0.49	0.12
**IGF2BP1**	0.75	0.13
**EFNB1**	0.77	0.09
**EFNA3**	0.78	0.19
**NOTCH1**	0.81	0.05
**TAZ**	0.89	0.09
**Top upregulated genes**	**Fold Change**	**STDEV**
**MPP4**	2.77	0.15
**CPA4**	2.28	0.49
**DNER**	2.04	0.18
**SEMA3C**	1.95	0.06
**PTPRN**	1.72	0.15
**TNFRSF12A**	1.61	0.33

Fold change in expression is determined by qRT-PCR validation of 3 biological replicates (p < 0.05).

**Fig 4:**
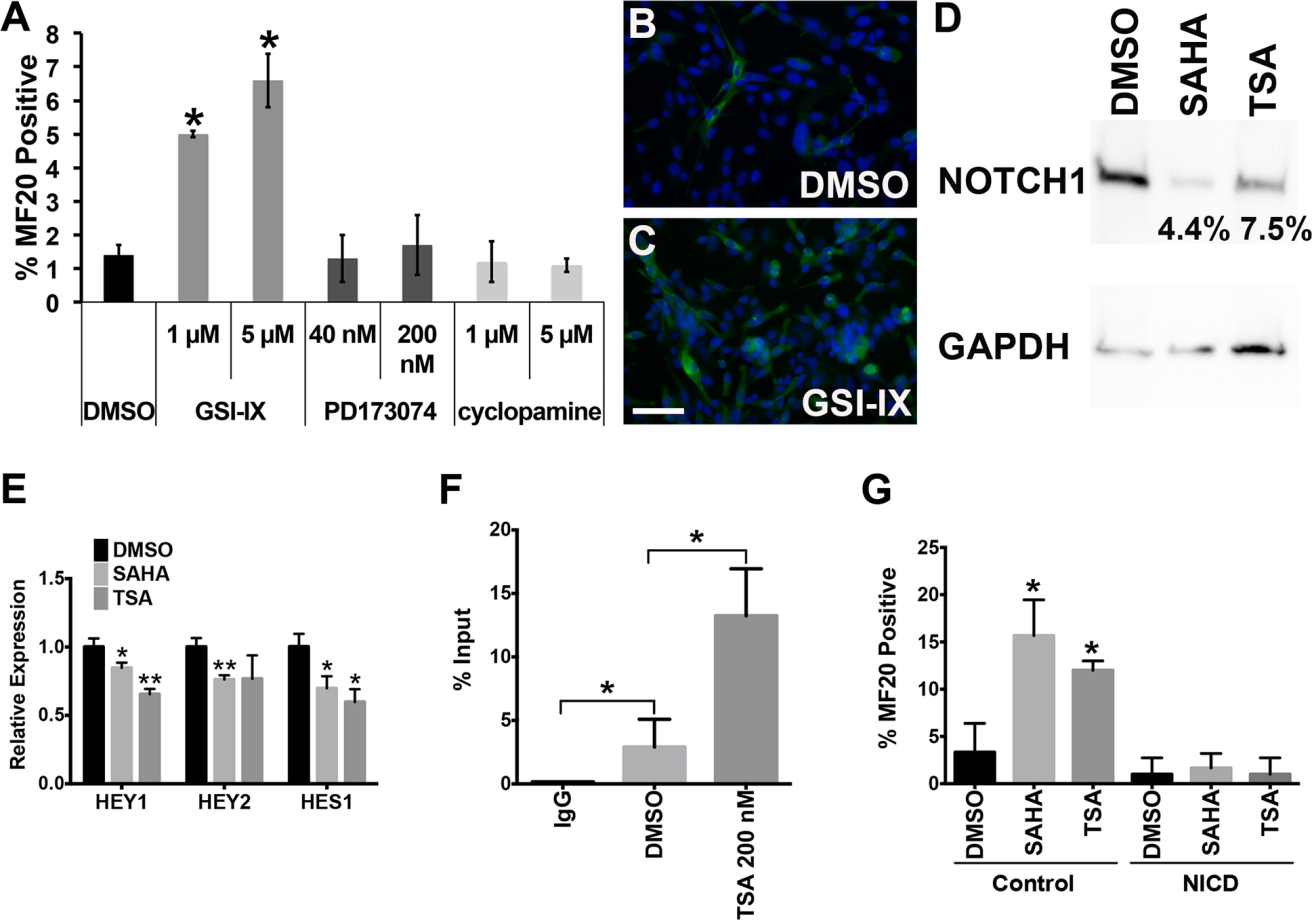
Aberrant NOTCH1-mediated signaling activity is essential for inhibiting myogenic differentiation of ERMS. (A) Summary of MF20 IF of RD cells treated with two different doses of GSI-IX, PD173074, or cyclopamine. (B-C) Representative MF20 IF images of RD cells treated with DMSO (B) and 20 μM GSI-IX (C). Scale bar = 20 μm. (D) Western blot of NOTCH1 expression in RD cells treated with DMSO, 200 nM TSA, or 1 μM SAHA. Each band intensity was normalized to GAPDH loading control. Percent intensity relative to DMSO (vehicle) treatment is shown. (E) Quantitative RT-PCR analysis of NOTCH1 pathway downstream targets, *HEY1*, *HEY2* and *HES1* in RD cells treated with DMSO, 200 nM TSA, or 1 μM SAHA. (F) ChIP assay summary showing differential binding of acetyl-histone H3 (Lys9) at the *NOTCH1* promoter in RD cells treated with DMSO or 200 nM TSA. Rabbit IgG was used as a negative control for chromatin immunoprecipitation. (G) Summary of MF20 IF of control GFP-overexpressing and NICD-overexpressing RD cells treated with DMSO, 200 nM TSA or 1 μM SAHA. Error bar in each graph indicates standard deviation of technical triplicates. Brackets in each group are used to indicate comparison groups. * indicates p < 0.05. ** indicates p < 0.01.

To determine which of the downregulated pathways upon TSA treatment functions downstream of HDAC activity to exert oncogenic or tumor suppressive effects in ERMS, we utilized pathway-specific small molecule inhibitors. Treatment of human ERMS cells with inhibitors specific for the Notch, Hedgehog and FGF-mediated pathways resulted in significant suppression of tumor growth *in vitro* (S3A–S3C Fig). However, only treatment with the inhibitor of the Notch pathway, GSI-IX (DAPT), resulted in significant induction of myogenic differentiation of RD and 381T cells, with at least 5-fold increase compared to treatment with vehicle control (results for treatment in RD cells shown in Fig 4A–4C). To validate the role of the Notch pathway in inhibiting myogenic differentiation, we showed that knockdown of NOTCH1 by gene-specific shRNA resulted in significant myogenic differentiation of ERMS cells (S3D and S3E Fig). Upon TSA and SAHA treatment, NOTCH1 mRNA and protein expression levels were reduced (Table 4 and Fig 4D) and downstream target genes, HEY1, HEY2, and HES1, were also downregulated in ERMS cells (Fig 4E). Interestingly, there was enriched binding of acetyl-histone H3 (Lys9) on the *NOTCH1* promoter in ERMS cells treated with TSA or SAHA (Fig 4F and S3F Fig), suggesting that histone hyperacetylation does not necessarily correlate with active transcription and/or other repressive events are present to suppress transcription of *NOTCH1* upon TSA or SAHA treatment. To assess the specificity of the Notch pathway as a downstream effector of HDAC-induced myogenic differentiation arrest, we showed that overexpression of Notch Intracellular Domain (NICD), which constitutively activates downstream signaling of the Notch pathway, reversed the myogenic differentiation phenotype resulting from TSA or SAHA treatment (Fig 4G and S3G Fig). Together, our results indicate that the Notch pathway is directly regulated by HDACs to suppress myogenic differentiation in ERMS.

### EFNB1 is regulated by HDACs to modulate migratory behavior of ERMS cells

**Fig 5:**
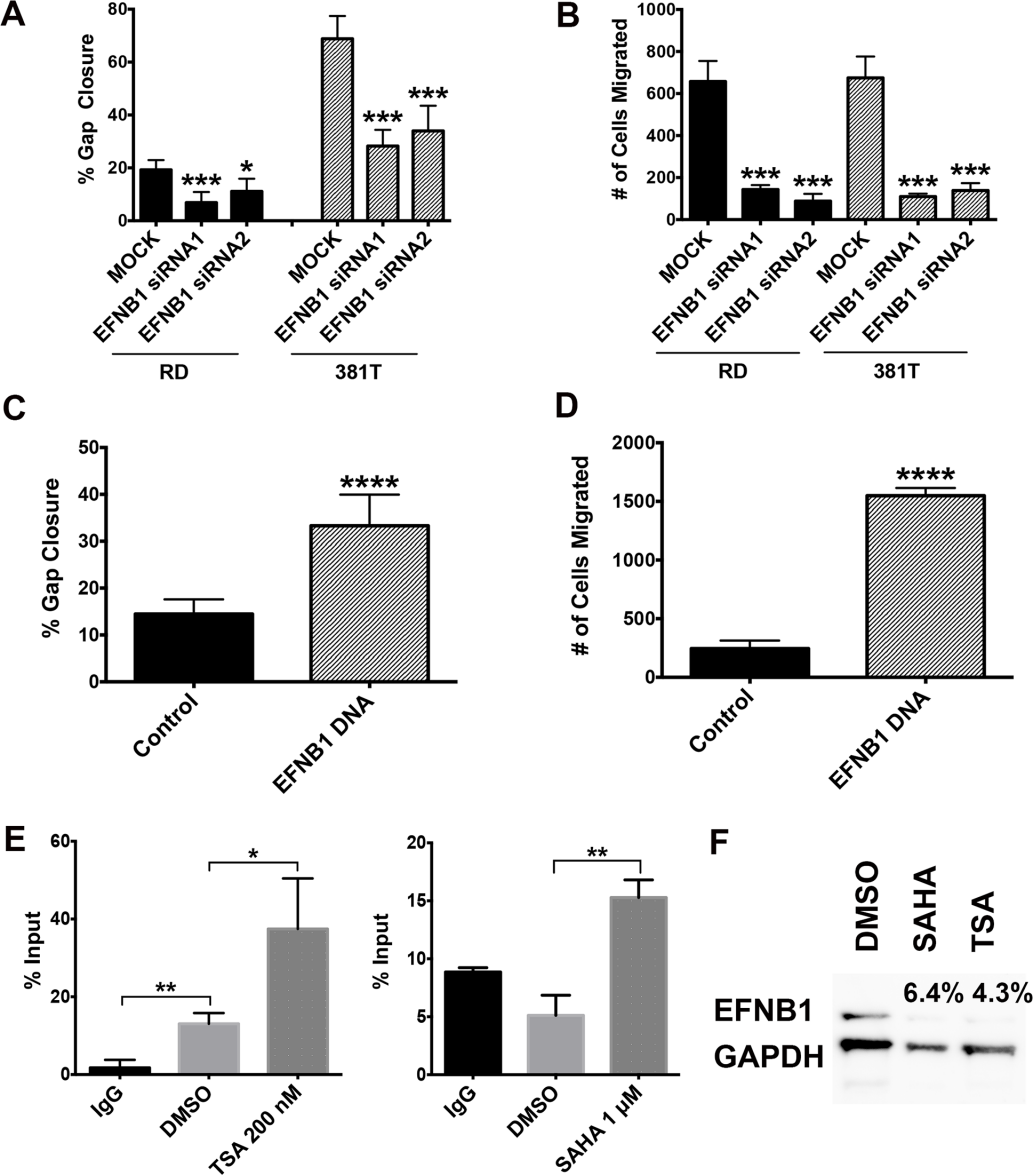
EFNB1 is essential for modulating the migratory capacity of ERMS cells. Summary of (A) scratch assays and (B) transwell migration assays on RD and 381T cells with EFNB1 knockdown by two independent siRNAs. Summary of (C) scratch assays and (D) transwell migration assays performed on control GFP-overexpressing and EFNB1-overexpressing RD cell lines. (E) ChIP assays showing differential binding of acetyl-histone H3 (Lys9) on *EFNB1* promoter in RD cells treated with DMSO, 200 nM TSA, or 1μM SAHA. Rabbit IgG was used as a negative control for chromatin immunoprecipitation. (F) Western blot assessing EFNB1 protein expression level in RD cells treated with DMSO, 200 nM TSA or 1 μM SAHA. Percent intensity relative to DMSO (vehicle) treatment is shown. GAPDH was used as a loading control.

Expression of Ephrin-A3 (*EFNA3*) and Ephrin-B1 (*EFNB1*) were downregulated in ERMS cells upon TSA treatment (Table 4). The Eph family of receptor tyrosine kinases and their membrane-associated ephrin ligands are known for their importance in a variety of developmental processes and in tissue morphogenesis [22–24]. In many cancer types, the Eph receptors and ephrins have also been shown to promote tumor cell invasion and metastasis [25–27]. To determine if EFNA3 and EFNB1 are essential for regulating the migratory capacity of ERMS cells, we performed loss-of-function studies using two independent siRNAs targeted against EFNA3 and EFNB1. We first showed that targeting EFNB1 and EFNA3 by gene-specific siRNAs resulted in effective gene knockdown by quantitative RT-PCR and/or Western blot analysis (S4A and S4B Fig). As assessed by scratch assay, RD or 381T cells transfected with EFNB1 siRNA showed a significant reduction in the percentage of gap closure in comparison to a mock control (Fig 5A). By contrast, knockdown of EFNA3 did not affect the percent of gap closure in the scratch assay (S4C Fig). We subsequently validated the effect of EFNB1 knockdown on the migratory capacity of ERMS cells using a separate transwell migration assay (Fig 5B). EFNB1 knockdown did not affect proliferation or apoptosis of the ERMS cells (S4D and S4E Fig), indicating that the reduced migratory capacity of the ERMS cells is not secondary to altered cell growth or increased cell death. To assess the effect of EFNB1 gain-of-function, a stable line of RD cells overexpressing EFNB1 was generated. Overexpression of EFNB1 mRNA and protein was confirmed by quantitative RT-PCR and Western blot analysis (S4F Fig). In contrast to GFP-expressing control cells, RD cells overexpressing EFNB1 showed at least 3-fold increase in their migratory capacity in both scratch and transwell assays (Fig 5C and 5D). Finally, by ChIP analysis, TSA or SAHA-treatment resulted in enriched binding of acetylated histones on the *EFNB1* promoter region (Fig 5E). EFNB1 protein expression level was reduced upon TSA or SAHA treatment (Fig 5F). Taken together, these data suggest that HDACs regulate EFNB1 to modulate the migratory capacity of ERMS cells. Results from our functional assays *in vitro* also suggest that Ephrin-B1 does not play a role in other HDAC inhibitor-induced effects such as abnormal cell cycle progression or differentiation.

### EFNB1 is differentially expressed in ERMS in comparison to ARMS patients

**Fig 6:**
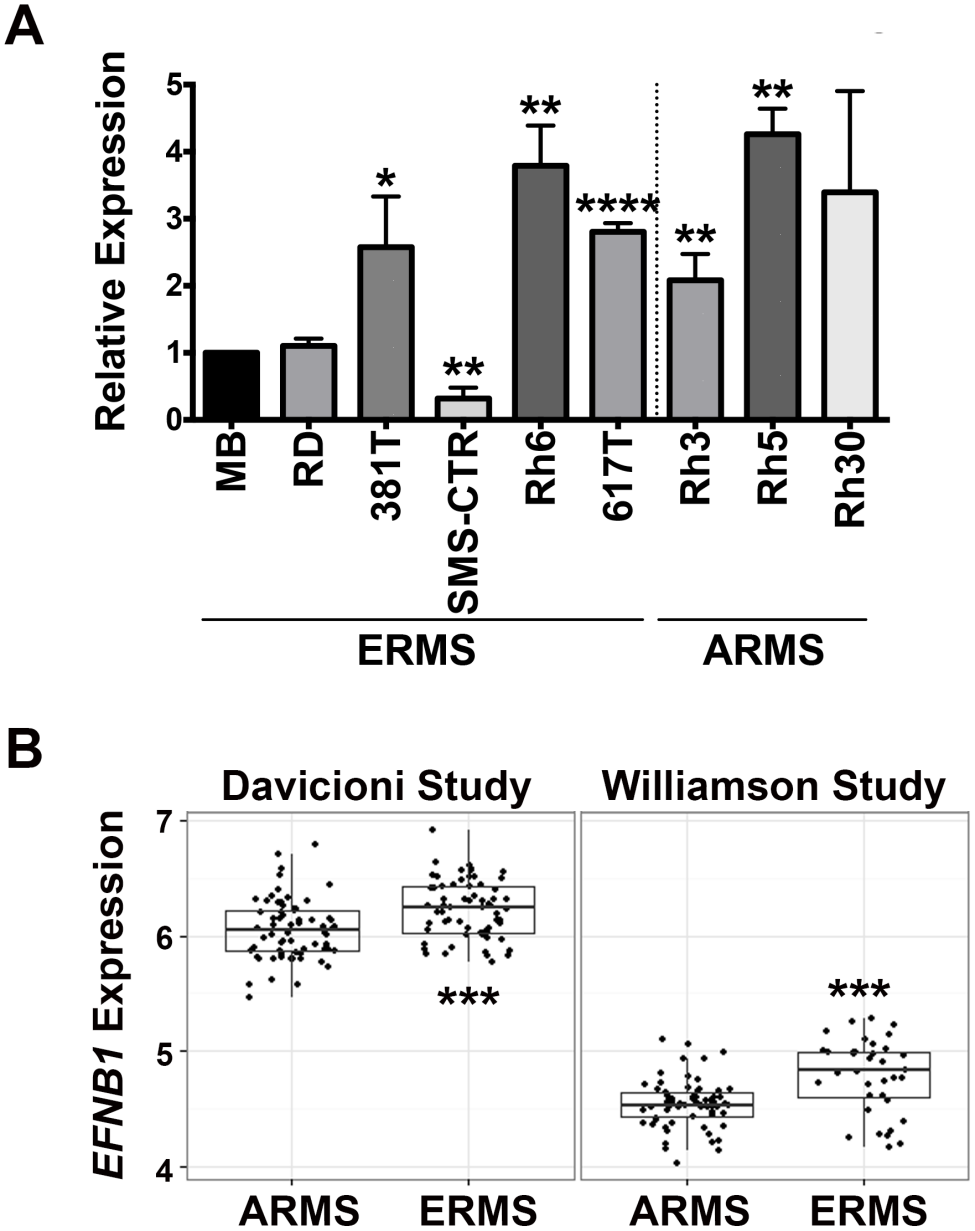
*EFNB1* expression is differentially expressed in ERMS patients compared to ARMS patients. (A) Quantitative RT-PCR showing expression of *EFNB1* in a panel of ERMS (RD, 381T, SMS-CTR, Rh6, and 617T) and ARMS (Rh3, Rh5, and Rh30) cell lines and myoblasts (MB) as a reference for normal muscle. (B) Comparison of *EFNB1* expression levels between ERMS (n = 66 for the Davicioni study; n = 36 for the Williamson study) and ARMS (n = 65 for the Davicioni study; n = 65 for the Williamson study) subtypes. * indicates p < 0.05. ** indicates p < 0.01. *** indicates p < 0.001. **** indicates p < 0.0001.

In our study, EFNB1 regulated the migratory behavior of ERMS cells, suggesting its potential role in affecting the invasive potential and therefore possibly the clinical prognosis of ERMS. We first showed that there was variable up-regulation of *EFNB1* expression in 3 of 5 ERMS cell lines tested (381T, Rh6 and 617T) and 2 of 3 ARMS cell lines tested (Rh3 and Rh5), compared to normal human myoblasts (Fig 6A). To assess the correlation of *EFNB1* expression in human ERMS patients with clinical survival data, we analyzed *EFNB1* expression and associated clinical data of 160 RMS patients from the study by Davicioni and colleagues [28] as well as 101 RMS patients from the study by Williamson and colleagues [29]. Using a two-sample t-test, the expression level of *EFNB1* was significantly increased in the ERMS cohort in comparison to the ARMS cohort in both studies (p < 0.001, Fig 6B). Complete survival data are available for 124 patients in the Davicioni study and 101 patients in the Williamson study. Within each subtype, we used Cox regression to analyze the relationship between survival and level of *EFNB1* expression (split at the median value and defined as high and low). There is no statistically significant difference in overall survival when comparing high-*EFNB1* expressing vs low-*EFNB1* expressing cohorts in both studies (S5 Fig). However, this analysis was limited by the low number of death events (11 deaths in the Davicioni study and 34 deaths in the Williamson study). As *EFNB1* expression is differentially increased in ERMS patients in comparison to ARMS patients, *EFNB1* likely serves a unique role in the pathogenesis of ERMS.

## Discussion

By characterizing the effects of two pan-HDAC inhibitors (trichostatin A and SAHA) *in vitro* and *in vivo*, this study has revealed diverse and essential roles of HDACs in driving the tumorigenesis of ERMS. Through differential expression profiling studies and pathway analysis, we have demonstrated downregulation of key oncogenic pathways including Hedgehog, FGF and Notch pathways in ERMS cells upon treatment of HDAC inhibitors. Using gain-of-function and loss-of-function studies, we further showed that HDACs regulate the differentiation and self-renewal status of ERMS through the Notch1-mediated pathway and migratory capacity through EFNB1-mediated pathway. Our findings suggest that HDAC function represents a major mechanism in driving ERMS tumor growth and progression.

Cancer cells show uncontrolled proliferation and fail to undergo tissue-specific differentiation. In differentiation therapy, tumor cells, including those that give rise to treatment resistance and disease recurrence, are induced to undergo terminal differentiation and thereby cease to proliferate. The success of differentiation therapy was first demonstrated in acute promyelocytic leukemia (APL), which was nearly universally lethal before the introduction of all-trans-retinoic acid-induced differentiation therapy. The cure rates now approach approximately 80% [30]. Differentiation therapy in solid tumors has shown clinical promise in the past decade [31–33]. Several pre-clinical *in vitro* and *in vivo* studies have demonstrated the efficacy of various agents in inducing tumor cell differentiation in sarcomas such as liposarcoma and osteosarcoma [34–36], suggesting that there is a therapeutic potential for differentiation therapy in sarcomas.

ERMS is pathologically characterized by an arrest in myogenic differentiation. In contrast to normal myogenesis, ERMS cells express myogenic regulatory factors such as MYOD1 and MYF5, but they fail to undergo terminal differentiation [37]. MYOD1 has been shown in ERMS cells to retain the ability to bind DNA, but is defective in activating myogenic target genes [38]. In this study, TSA and SAHA treatment induced myogenic differentiation of ERMS cells and dynamically regulated the expression levels of myogenic regulatory factors. The promoters of myogenic regulatory factors such as *MYOD1* and *MYOG* also showed differential histone acetylation patterns after HDAC inhibitor treatment, suggesting that aberrant epigenetic regulation by HDACs likely contributes to myogenic differentiation arrest in ERMS. In addition, HDAC inhibitor treatment reduced the self-renewal capacity of ERMS cells, suggesting that HDACs modulate the balance between differentiation and self-renewal of ERMS tumor cells. By contrast, TSA and SAHA treatment did not significantly alter the myogenic differentiation status of ARMS cells, suggesting that the myogenic arrest in ARMS is likely regulated by a different molecular mechanism. However, based on our *in vitro* (MF20 IF) and *in vivo* (quantitative FACS) results, TSA or SAHA treatment induced myogenic differentiation in a subset of ERMS cells, suggesting that other suppressive events need to be abrogated for complete induction of terminal myogenic differentiation in ERMS cells.

We subsequently showed by expression profiling that multiple oncogenic pathways such as Notch1, Hedgehog, and FGF-mediated pathways were downregulated in ERMS cells upon TSA treatment. The Hedgehog pathway has previously been shown to regulate self-renewal and differentiation of RMS. Work by Satheesa et al. has shown that inhibition of the Hedgehog signaling pathway either by overexpressing SUFU (Suppressor of Fused) or knocking down Smoothened (SMO) decreases self-renewal capacity and increases myogenic differentiation of ERMS cells [39]. In a mouse model of RMS with *Patched1* (*Ptch1*) haploinsufficency, Gli1 and Gli2, the Hedgehog-regulated transcriptional factors, have been shown to suppress myogenesis of RMS by repressing the capacity of MyoD to activate transcription [40]. In addition, calcitriol, the active form of vitamin D3, inhibits Hedgehog signaling to induce differentiation of RMS derived from mice with *Ptch1* mutations [41]. In our study, inhibition of Hedgehog signaling by cyclopamine (up to 5 μM) did not induce significant myogenic differentiation of ERMS cells, suggesting variable treatment response among human ERMS cell lines. By contrast, inhibition of NOTCH1 in our study phenocopied the differentiation effect of TSA and SAHA, and overexpression of NICD abrogated the myogenic effect of TSA and SAHA. Overall, our results indicate that Notch represents a major pathway regulated by HDACs to modulate the differentiation status of ERMS cells. The studies by Raimondi et al. and De Salvo et al., have demonstrated that the inhibition of NOTCH3 signaling induces myogenic differentiation of both ARMS and ERMS cells by increasing p38 phosphorylation and p21^Cip1^ level [42], and the activated form of Notch3 increases RMS cell proliferation [43]. The findings indicate that the Notch gene family plays an important role in driving myogenic differentiation arrest in ERMS. Inhibition of HDAC function and downstream signaling pathways, including Notch1 and Notch3, promises an alternative therapeutic option (i.e. differentiation therapy) in the treatment of ERMS patients.

Our study also uncovered a novel role of Ephrin-B1 in regulating the migratory behavior of ERMS cells. The ephrin ligands and the Eph receptors are a cell surface-bound family of tyrosine kinases that have been shown to play an essential role in a variety of developmental and tissue morphogenetic events [24]. In particular, their complex roles in axon guidance are well characterized [23]. A subset of ephrins and Eph receptors has been shown to either inhibit or promote tumor growth and progression in several cancer types [44, 45]. In ERMS, high expression of PAX7 upregulates *EPHA3* and *EFNA1* to promote migration and invasiveness of tumor cells [46]. By contrast, suppression of *EPHA3* expression increases motility and migration in a subset of ERMS cell lines (TE671 and RD), indicating a context-dependent role of EPHA3 in either promoting or suppressing migratory behavior of ERMS cells. [47]. In a murine model of ARMS, Ephrin-B2 has dual signaling roles with its cognate ligand Ephrin-B2 to promote apoptosis and with PDGFRβ to promote proliferation [48]. In our study, we have demonstrated the important role of EFNB1 in regulating the migratory behavior of ERMS cells. *EFNB1* expression is downregulated in ERMS cells upon treatment with HDAC inhibitors, and *EFNB1* knockdown significantly reduces the migratory capacity of ERMS cells. Our findings suggest that aberrant regulation of EFNB1 activity by HDACs likely contributes to the invasive and metastatic potential of ERMS. Analysis of the RMS patients in the study by Davicioni et al. (2009) and Williamson et al. (2010) has also shown that *EFNB1* is differentially upregulated in ERMS in comparison to ARMS. Our study as well as previous studies have demonstrated *EFNB1* overexpression in a subset of ERMS cell lines and primary samples [49].Taken together, these results indicate that Ephrin-B1 expression likely plays an important role in promoting ERMS tumor progression by modulating the invasive behavior of tumor cells.

The current study identified distinct sets of upregulated and downregulated pathways in ERMS cells upon TSA treatment. There is inconsistent correlation between histone H3 (Lys9) hyperacetylation and gene transcription, as evidenced by the downregulation of *NOTCH1* and *EFNB1* genes after TSA treatment. Our findings suggest that HDAC inhibitors can alter the epigenetic state of the chromatin by hyperacetylation, but do not necessarily increase gene transcription. Similar observations have been made in other biological systems. For example, the study by Lopez-Atalaya et al. in adult mouse hippocampus demonstrated that TSA-induced histone hyperacetylation has a modest impact on global gene transcription, and histone acetylation changes upon TSA treatment do not always translate into significant changes in gene expression levels [50]. In human T cells, HDACs are frequently recruited to transcriptionally active regions of the genome to reset chromatin structure so as to prevent continuous gene activation, indicating that histone acetylation does not necessarily indicate active gene transcription [51]. The lack of correlation between histone acetylation status and gene transcription can also reflect molecular events independent of HDAC activity. For example, the HDAC inhibitors, SAHA and panobinostat, have previously been shown by Hedrick et al. to repress expression of oncogenic Sp transcription factors through a reactive oxygen species (ROS) dependent mechanism in RMS cells [52]. These findings are consistent with the previous finding of increased oxidative stress in RMS cell lines and xenografts treated with panobinostat [4]. Taken together, while our findings suggest that while HDAC activity plays an important role in regulating the tumor biology of ERMS, other histone acetylation-independent or repressive events induced by HDACs may also contribute to the tumorigenesis of ERMS.

In summary, this study has demonstrated the important roles of HDACs in ERMS pathogenesis by characterizing the anti-tumor effects of pan-HDAC inhibitors, trichostatin A and suberoylanilide hydroxamic acid (SAHA), in ERMS *in vitro* and *in vivo*. We have delineated Notch and Ephrin-B1 mediated pathways as essential downstream events of HDAC activity in the inhibition of myogenic differentiation and enhanced migratory capacity of ERMS cells, respectively. Aberrant epigenetic regulation by HDACs represents a major molecular mechanism driving tumorigenesis of ERMS. Potential druggable targets within the molecular network regulated by HDAC activity represent promising therapeutic options for the treatment of ERMS.

## Materials and Methods

### Animal protocol approval

Zebrafish tumor studies were approved by the Institutional Animal Care and Use Committee at University of Washington under the protocol 4330–01.

### Chemicals

Trichostatin A and suberoylanilide hydroxamic acid (SAHA) were obtained from Sigma Aldrich. Each compound was reconstituted in dimethylsulfoxide (DMSO) as 10–100 mM stocks. For chemical treatment of human cell lines, TSA or SAHA was diluted in 0.1% DMSO/growth medium.

### Cell lines, siRNA transfection, DNA electroporation and Western Analysis

The human RD, 381T, and 617T cell lines were obtained from ATCC cell biology collection (Manassas, Virginia). The human SMS-CTR, Rh6, Rh3, Rh5, Rh7, and Rh30 cell lines were gifts from Dr. Corinne Linardic at Duke University. Cells were seeded at a density of 2 x 10^5^ cells in 6-well plates in 2 ml of 10% FBS/1% Penicillin/Streptomycin/Glutamine in DMEM. 2.5 pg of gene-specific siRNA (purchased from Qiagen) were transfected into cells using RNAiMax lipofectamine transfection reagent (Life Technologies). For overexpression studies, the coding sequence (CDS) of *EFNB1* was cloned into a piggybac transposon vector driven by the *EF1α* promoter. Fusion of the *EFNB1* CDS with a T2A puromycin cassette enabled selection of stable cells. The overexpression vectors were purified with the Plasmid *Plus* Midi kit (Qiagen), and co-electroporated into the ERMS cell lines along with a Super PiggyBac transposase vector (ratio of 2.5:1; System Biosciences) using the Neon Transfection System (Life Technologies), with 2 pulses of 1150V for 30 milliseconds each. Stable ERMS cell lines expressing the constructs were established through puromycin selection for at least 2 weeks.

Total cell lysates from human ERMS cell lines were immunoblotted using primary antibodies against acetyl-histone H3 (Lys9) (1:1000; Active Motif), acetyl-histone H4 (Lys5) (1:1000; Active Motif), acetyl-histone H4 (Lys8) (1:1000; Cell Signaling), acetyl-histone H4 (Lys12) (1:1000; Active Motif) and GAPDH (1:2500; Cell Signaling). Zebrafish ERMS tumors were isolated by dissection, minced with a razor blade and resuspended in RIPA lysis buffer. Total cell lysates were immunoblotted using primary antibodies against acetyl-histone H3 (Lys9) (1:1000; Active Motif), acetyl-histone H4 (Lys5) (1:1000; Active Motif) and GAPDH (1:2500; Cell Signaling).

### Cell-based assays

ATP-based viability assay (CellTiter-Glo; Promega), EdU flow cytometry-based assay (Life Technologies), differentiation, sphere, scratch, and transwell migrations were performed as described previously [53] except for the following modifications. In the differentiation assay, cells were cultured in 2% horse serum and treated with TSA or SAHA for 3 days prior to fixation and immunostaining for MF20 (1:200; R&D Systems) and DAPI (1:500; Life Technologies). Images were taken using the Evos microscopy imaging system. In the sphere assay, 5000 cells per well were cultured in neurobasal medium supplemented with bFGF (1500 pg/μL), PDGF-AA (733 pg/μL), PDGF-BB (733 pg/μL) and EGF (5.2 pg/μL) in low-binding 24-well plates. Treatment with DMSO (vehicle), TSA (200 nM) and SAHA (1 μM) began on the day of plating and the number of spheres per well was scored 3 days post-treatment. For serial replating, spheres were harvested every 4 days and dissociated using StemPro Accutase (Life Technologies). The cells were resuspended in the neurobasal medium supplemented with growth factors and replated into a new low-binding plate with continued drug treatment. The number of spheres per well was scored 3 days post-replating. In the scratch assay, treatment of human ERMS with TSA or SAHA began 6 hours prior to scratch. Each treatment condition was performed in duplicates. Bright field images of pre- and 18 hours post-scratch were taken using the Evos microscopy imaging system (Life Technologies). In the transwell migration assay, after a 22-hour migration period, three random fields of the membranes containing migrated cells were imaged using the Nikon stereoscope (SMZ18, 3x magnification) and counted using ImageJ software. A Student’s t-test was performed to assess differences between the control and experimental groups.

### Chromatin Immunoprecipitation

RD and 381T cells cultured in growth medium were cross-linked with 1% formaldehyde 24 hours following chemical (TSA or DMSO) treatment or EFNB1 siRNA transfection. Chromatin immunoprecipitation was performed using the MAGnify Chromatin Immunoprecipitation system (Life Technologies) with the following modifications. Chromatin was initially digested with 2–3 units of micrococcal nuclease per million cells (New England Biolabs) for 15 minutes prior to sonication using the Misonix S-4000 sonicator (Amp 2, 25 cycles). Sheared chromatin fragments were in the range of 300–500 bp as detected on an ethidium bromide-stained 2% agarose gel. 1 μg of anti-acetyl-histone H3 (Lys9) (Active Motif) was used in binding chromatin. Rabbit IgG antibody supplied by the kit was used as a negative control in the ChIP assay. Purified DNA fragments were used in quantitative PCR (95C for 2 min, 95C for 15 sec, 63C for 30 sec, 72C for 30 sec, 40 cycles) using a Rotor-gene 3000 (Qiagen) to assess for enrichment of gene-specific promoter regions. Primers used in the ChIP assay are listed in S1 Table.

### Chemical treatment of zebrafish with ERMS for tumor growth and limiting dilution assays

Six-week old CG1 syngeneic fish were transplanted with 3x10^5^ unsorted tumor cells arising from Tg(*myf5*:GFP, *mylz2*:mCherry) ERMS from CG1 strain fish [54]. Engrafted animals were treated at 7 days post-transplantation with 1 μM of TSA or 50 μM of SAHA and vehicle control (DMSO) for 5 days (including one 24-hr drug holiday). Tumor volume was assessed by imaging animals pre- and post-treatment. Tumor volume was calculated by multiplying tumor area by fluorescent intensity using ImageJ. A Student’s t-test was performed to assess differences between tumor size in the control and experimental groups. For limiting dilution experiments, the *myf5*:GFP^+^/*mylz2*:mCherry^−^tumor cell subpopulation was sorted by FACS prior to transplanting at limiting dilutions of 10^3^, 10^2^ and 10 cells into six-week old CG1 syngeneic fish. Unsorted bulk tumor cells were transplanted at limiting dilutions of 10^4^, 10^3^ and 10^2^ cells. Self-renewal frequency was determined by the extreme limiting dilution analysis (ELDA) algorithm [55].

Tumor-bearing zebrafish were euthanized at or before tumor endpoint criteria, defined as 1) tumor invasion from trunk skeletal muscle into adjacent tissues/organs or 2) tumor grows to 50% of host body mass. No tumor exceeded 50% of host body mass. Zebrafish were euthanized by immersion in an overdose of neutral buffered Tricaine followed by decapitation or icing.

### Analysis of human clinical data

Clinical and expression data of RMS patients were obtained from the supplemental materials of the study by Davicioni et al. (2009) and Williamson et al. (2010). A Cox regression model with the assumption of a linear relationship between the expression level and the hazard ratio was used to generate the survival curves for ERMS and ARMS cohorts.

### Microarray analysis

RD and 381T cells treated with DMSO and TSA (200 nM) were harvested 24 hours post-treatment. RNA was isolated from the cells using the Qiagen RNeasy Kit. Integrity of RNA samples was assessed with an Agilent 2100 Bioanalyzer (Agilent Technologies Inc., Santa Clara, CA). Processing of the RNA samples that passed quality control was performed according to the standard Affymetrix GeneChip Whole Transcript Sense Target labeling protocol. The arrays were scanned with an Affymetrix GeneChip® 3000 scanner. Image generation and feature extraction were performed using Affymetrix GeneChip Command Console Software. The data at the transcript level were summarized using the RMA algorithm, as implemented in the Bioconductor oligo package [56]. We filtered out all control probesets, as well as those probesets that appear to be either not expressed, or at such a low level that noise dominates the signal. After filtering there were 19,211 probesets that remained. We then fit a weighted analysis of variance (ANOVA) model [57] and made comparisons using empirical Bayes adjusted contrasts, using the Bioconductor limma package [55].

Microarray data can be accessed at NCBI Gene Expression Omnibus (GEO) as series GSE74970.

## Supporting Information

S1 Fig SAHA exerted anti-tumor effects in ERMS by reducing tumor growth and altering cell cycle progression. (A-B) CellTiter-Glo viability assays on RD cells treated with DMSO, 3 doses of TSA (A) or 3 doses of SAHA (B). (C-D) Cell cycle analysis of RD and 381T cells treated with TSA (C) or SAHA (D). (E-F) Annexin V analysis of RD, 381T and SMS-CTR cells treated with TSA (E) or SAHA (F). Each error bar indicates standard deviation of technical triplicates.
(TIF)

S2 Fig SAHA induced differentiation and reduced self-renewal of ERMS cells. (A-F) Quantitative RT-PCR of myogenic genes *MYOD1*, *MYOG*, and *MYH1* after 24 hour treatment with either DMSO or 200 nM TSA (A-C) or 1 μM SAHA (D-F). (G-H) Representative bright field images from sphere assay of ERMS cells treated with DMSO or 1 μM SAHA. (I-J) Serial replating of RD and 381T spheres treated with 1 μM SAHA. Each error bar denotes standard deviation of experimental triplicate. * indicates p < 0.05. ** indicates p < 0.01.
(TIF)

S3 Fig NOTCH1 pathway enhanced tumor growth and inhibited myogenic differentiation. (A-C) Summary of CellTiter-Glo viability assays of RD cells treated with DMSO or GSI-IX (A), PD173074 (B), or cyclopamine (C). Fold change in ATP luminescence signal intensity over 4 days is shown. Error bars indicate standard deviation of technical triplicates. (D-E) Representative images of MF20 immuofluorescence of RD cells harboring control shRNA (D) and NOTCH1 shRNA (E). Quantitation of percent MF20+ cells including standard deviation is shown on each panel. Scale bar indicates 20 μm. (F) ChIP assay showing differential binding of acetyl-histone H3 (Lys9) on *NOTCH1* promoter in RD cells treated with DMSO or 1 μM SAHA. Rabbit IgG was used as a negative control for chromatin immunoprecipitation. (G) Summary of MF20 IF of control GFP-overexpressing and NICD-overexpressing 381T cells treated with DMSO, 200 nM TSA or 1 μM SAHA. Error bar in each panel indicates standard deviation of experimental triplicates. * indicates p < 0.05. ** indicates p < 0.01. *** indicates p < 0.001.
(TIF)

S4 Fig EFNB1 but not EFNA3 affected migratory capacity of ERMS cells. (A) Quantitative RT-PCR showing effective knockdown of *EFNB1* and *EFNA3* mRNA expression using 2 independent gene-specific siRNAs. Levels are shown in comparison to mock-treated samples. (B) Western blot analysis showing effective knockdown of EFNB1 protein level in RD and 381T cells by *EFNB1* siRNA. Each band intensity was normalized to Lamin B1 (LMNB1) loading control. % knockdown of EFNB1 relative to mock treatment is indicated. (C) Summary of scratch assay performed on RD and 381T cells with EFNA3 knockdown by 2 independent siRNAs. (D) EdU flow cytometry-based assay to assess proliferation rate of RD and 381T cells with EFNB1 knockdown by 2 independent siRNAs. (E) Annexin V flow cytometry-based assay to assess the extent of apoptosis in RD and 381T cells with EFNB1 knockdown by 2 independent siRNAs. (F) Quantitative RT-PCR confirming increased expression of *EFNB1* mRNA in the overexpression cell line. (G) Western blot analysis confirming increased expression of EFNB1 protein in the overexpression cell line. Each band intensity was normalized to GAPDH loading control. Fold expression compared to control GFP-overexpressing cell line is indicated. Error bar in each panel indicates standard deviation of experimental triplicates. ** indicates p < 0.01. *** indicates p < 0.001. **** indicates p < 0.0001.
(TIF)

S5 Fig Correlation of EFNB1 expression with overall survival in ERMS and ARMS patients. Kaplan-Meier curves comparing the probability of survival between levels of *EFNB1* expression within ERMS and ARMS patients (A-B) Results for the Davicioni study: ERMS (n = 62, 11 deaths) and ARMS (n = 62, 27 deaths). (C-D) Results for the Williamson study: ERMS (n = 36, 5 deaths) and ARMS (n = 65, 29 deaths). Red: high *EFNB1* expression. Blue: low *EFNB1* expression.
(TIF)

S1 Table Primers used in quantitative PCR. (PDF)

## References

[1] Bridge JA, Liu J, Qualman SJ, Suijkerbuijk R, Wenger G, Zhang J, et al Genomic gains and losses are similar in genetic and histologic subsets of rhabdomyosarcoma, whereas amplification predominates in embryonal with anaplasia and alveolar subtypes. Genes Chromosomes Cancer. 2002;33(3):310–21. Epub 2002/01/25. 10.1002/gcc.10026 [pii]. PubMed PMID: . 11807989

[2] Goldstein M, Meller I, Issakov J, Orr-Urtreger A. Novel genes implicated in embryonal, alveolar, and pleomorphic rhabdomyosarcoma: a cytogenetic and molecular analysis of primary tumors. Neoplasia. 2006;8(5):332–43. Epub 2006/06/23. 10.1593/neo.05829 16790082 PMC1592451

[3] Missiaglia E, Selfe J, Hamdi M, Williamson D, Schaaf G, Fang C, et al Genomic imbalances in rhabdomyosarcoma cell lines affect expression of genes frequently altered in primary tumors: an approach to identify candidate genes involved in tumor development. Genes Chromosomes Cancer. 2009;48(6):455–67. Epub 2009/02/25. 10.1002/gcc.20655 . 19235922

[4] Chen X, Stewart E, Shelat AA, Qu C, Bahrami A, Hatley M, et al Targeting oxidative stress in embryonal rhabdomyosarcoma. Cancer Cell. 2013;24(6):710–24. 10.1016/j.ccr.2013.11.002 24332040 PMC3904731

[5] Chen Y, Takita J, Hiwatari M, Igarashi T, Hanada R, Kikuchi A, et al Mutations of the PTPN11 and RAS genes in rhabdomyosarcoma and pediatric hematological malignancies. Genes Chromosomes Cancer. 2006;45(6):583–91. Epub 2006/03/07. 10.1002/gcc.20322 . 16518851

[6] Paulson V, Chandler G, Rakheja D, Galindo RL, Wilson K, Amatruda JF, et al High-resolution array CGH identifies common mechanisms that drive embryonal rhabdomyosarcoma pathogenesis. Genes Chromosomes Cancer. 2011;50(6):397–408. Epub 2011/03/18. 10.1002/gcc.20864 . 21412928

[7] Shern JF, Chen L, Chmielecki J, Wei JS, Patidar R, Rosenberg M, et al Comprehensive genomic analysis of rhabdomyosarcoma reveals a landscape of alterations affecting a common genetic axis in fusion-positive and fusion-negative tumors. Cancer Discov. 2014;4(2):216–31. 10.1158/2159-8290.CD-13-0639 . 24436047 PMC4462130

[8] Chen EY, DeRan MT, Ignatius MS, Grandinetti KB, Clagg R, McCarthy KM, et al Glycogen synthase kinase 3 inhibitors induce the canonical WNT/beta-catenin pathway to suppress growth and self-renewal in embryonal rhabdomyosarcoma. Proc Natl Acad Sci U S A. 2014;111(14):5349–54. 10.1073/pnas.1317731111 24706870 PMC3986146

[9] Khan O, La Thangue NB. HDAC inhibitors in cancer biology: emerging mechanisms and clinical applications. Immunol Cell Biol. 2012;90(1):85–94. Epub 2011/11/30. 10.1038/icb.2011.100 . 22124371

[10] West AC, Johnstone RW. New and emerging HDAC inhibitors for cancer treatment. J Clin Invest. 2014;124(1):30–9. Epub 2014/01/03. 10.1172/JCI69738 24382387 PMC3871231

[11] Jain S, Zain J. Romidepsin in the treatment of cutaneous T-cell lymphoma. J Blood Med. 2011;2:37–47. 10.2147/JBM.S9649 22287862 PMC3262342

[12] Mann BS, Johnson JR, Cohen MH, Justice R, Pazdur R. FDA approval summary: vorinostat for treatment of advanced primary cutaneous T-cell lymphoma. Oncologist. 2007;12(10):1247–52. 10.1634/theoncologist.12-10-1247 . 17962618

[13] Blattmann C, Oertel S, Ehemann V, Thiemann M, Huber PE, Bischof M, et al Enhancement of radiation response in osteosarcoma and rhabdomyosarcoma cell lines by histone deacetylase inhibition. Int J Radiat Oncol Biol Phys. 2010;78(1):237–45. 10.1016/j.ijrobp.2010.03.010 . 20646843

[14] Kutko MC, Glick RD, Butler LM, Coffey DC, Rifkind RA, Marks PA, et al Histone deacetylase inhibitors induce growth suppression and cell death in human rhabdomyosarcoma in vitro. Clin Cancer Res. 2003;9(15):5749–55. PubMed PMID: . 14654560

[15] Abraham J, Nunez-Alvarez Y, Hettmer S, Carrio E, Chen HI, Nishijo K, et al Lineage of origin in rhabdomyosarcoma informs pharmacological response. Genes Dev. 2014;28(14):1578–91. 10.1101/gad.238733.114 25030697 PMC4102765

[16] Walter D, Satheesha S, Albrecht P, Bornhauser BC, D'Alessandro V, Oesch SM, et al CD133 positive embryonal rhabdomyosarcoma stem-like cell population is enriched in rhabdospheres. PLoS One. 2011;6(5):e19506 10.1371/journal.pone.0019506 21602936 PMC3094354

[17] Ignatius MS, Chen E, Elpek NM, Fuller AZ, Tenente IM, Clagg R, et al In vivo imaging of tumor-propagating cells, regional tumor heterogeneity, and dynamic cell movements in embryonal rhabdomyosarcoma. Cancer Cell. 2012;21(5):680–93. 10.1016/j.ccr.2012.03.043 22624717 PMC3381357

[18] Langenau DM, Keefe MD, Storer NY, Guyon JR, Kutok JL, Le X, et al Effects of RAS on the genesis of embryonal rhabdomyosarcoma. Genes Dev. 2007;21(11):1382–95. 10.1101/gad.1545007 17510286 PMC1877750

[19] Belyea BC, Naini S, Bentley RC, Linardic CM. Inhibition of the Notch-Hey1 axis blocks embryonal rhabdomyosarcoma tumorigenesis. Clin Cancer Res. 2011;17(23):7324–36. 10.1158/1078-0432.CCR-11-1004 21948088 PMC3241994

[20] Gorlin RJ. Nevoid basal cell carcinoma (Gorlin) syndrome. Genet Med. 2004;6(6):530–9. . 15545751 10.1097/01.gim.0000144188.15902.c4

[21] Hatley ME, Tang W, Garcia MR, Finkelstein D, Millay DP, Liu N, et al A mouse model of rhabdomyosarcoma originating from the adipocyte lineage. Cancer Cell. 2012;22(4):536–46. 10.1016/j.ccr.2012.09.004 23079662 PMC3479681

[22] Davy A, Aubin J, Soriano P. Ephrin-B1 forward and reverse signaling are required during mouse development. Genes Dev. 2004;18(5):572–83. 10.1101/gad.1171704 15037550 PMC374238

[23] Egea J, Klein R. Bidirectional Eph-ephrin signaling during axon guidance. Trends Cell Biol. 2007;17(5):230–8. 10.1016/j.tcb.2007.03.004 . 17420126

[24] Pasquale EB. Eph receptor signalling casts a wide net on cell behaviour. Nat Rev Mol Cell Biol. 2005;6(6):462–75. 10.1038/nrm1662 . 15928710

[25] Gao Q, Liu W, Cai J, Li M, Gao Y, Lin W, et al EphB2 promotes cervical cancer progression by inducing epithelial-mesenchymal transition. Hum Pathol. 2014;45(2):372–81. 10.1016/j.humpath.2013.10.001 . 24439224

[26] Kandouz M. The Eph/Ephrin family in cancer metastasis: communication at the service of invasion. Cancer Metastasis Rev. 2012;31(1–2):353–73. 10.1007/s10555-012-9352-1 . 22549394

[27] Petty A, Myshkin E, Qin H, Guo H, Miao H, Tochtrop GP, et al A small molecule agonist of EphA2 receptor tyrosine kinase inhibits tumor cell migration in vitro and prostate cancer metastasis in vivo. PLoS One. 2012;7(8):e42120 10.1371/journal.pone.0042120 22916121 PMC3419725

[28] Davicioni E, Anderson MJ, Finckenstein FG, Lynch JC, Qualman SJ, Shimada H, et al Molecular classification of rhabdomyosarcoma—genotypic and phenotypic determinants of diagnosis: a report from the Children's Oncology Group. Am J Pathol. 2009;174(2):550–64. 10.2353/ajpath.2009.080631 19147825 PMC2630563

[29] Williamson D, Missiaglia E, de Reynies A, Pierron G, Thuille B, Palenzuela G, et al Fusion gene-negative alveolar rhabdomyosarcoma is clinically and molecularly indistinguishable from embryonal rhabdomyosarcoma. Journal of clinical oncology: official journal of the American Society of Clinical Oncology. 2010;28(13):2151–8. 10.1200/JCO.2009.26.3814 . 20351326

[30] Stone RM, Maguire M, Goldberg MA, Antin JH, Rosenthal DS, Mayer RJ. Complete remission in acute promyelocytic leukemia despite persistence of abnormal bone marrow promyelocytes during induction therapy: experience in 34 patients. Blood. 1988;71(3):690–6. PubMed PMID: . 3422828

[31] Azzi S, Bruno S, Giron-Michel J, Clay D, Devocelle A, Croce M, et al Differentiation therapy: targeting human renal cancer stem cells with interleukin 15. J Natl Cancer Inst. 2011;103(24):1884–98. 10.1093/jnci/djr451 . 22043039

[32] Chien AJ, Moore EC, Lonsdorf AS, Kulikauskas RM, Rothberg BG, Berger AJ, et al Activated Wnt/beta-catenin signaling in melanoma is associated with decreased proliferation in patient tumors and a murine melanoma model. Proc Natl Acad Sci U S A. 2009;106(4):1193–8. 10.1073/pnas.0811902106 19144919 PMC2626610

[33] Rephaeli A, Blank-Porat D, Tarasenko N, Entin-Meer M, Levovich I, Cutts SM, et al In vivo and in vitro antitumor activity of butyroyloxymethyl-diethyl phosphate (AN-7), a histone deacetylase inhibitor, in human prostate cancer. Int J Cancer. 2005;116(2):226–35. 10.1002/ijc.21030 . 15800932

[34] Charytonowicz E, Terry M, Coakley K, Telis L, Remotti F, Cordon-Cardo C, et al PPARgamma agonists enhance ET-743-induced adipogenic differentiation in a transgenic mouse model of myxoid round cell liposarcoma. J Clin Invest. 2012;122(3):886–98. 10.1172/JCI60015 22293175 PMC3287226

[35] Demetri GD, Fletcher CD, Mueller E, Sarraf P, Naujoks R, Campbell N, et al Induction of solid tumor differentiation by the peroxisome proliferator-activated receptor-gamma ligand troglitazone in patients with liposarcoma. Proc Natl Acad Sci U S A. 1999;96(7):3951–6. PubMed PMID: 10097144 10.1073/pnas.96.7.3951 PMC22401

[36] Haydon RC, Zhou L, Feng T, Breyer B, Cheng H, Jiang W, et al Nuclear receptor agonists as potential differentiation therapy agents for human osteosarcoma. Clin Cancer Res. 2002;8(5):1288–94. PubMed PMID: . 12006550 PMC4527755

[37] Tapscott SJ, Thayer MJ, Weintraub H. Deficiency in rhabdomyosarcomas of a factor required for MyoD activity and myogenesis. Science. 1993;259(5100):1450–3. PubMed PMID: . 8383879 10.1126/science.8383879

[38] Yang Z, MacQuarrie KL, Analau E, Tyler AE, Dilworth FJ, Cao Y, et al MyoD and E-protein heterodimers switch rhabdomyosarcoma cells from an arrested myoblast phase to a differentiated state. Genes Dev. 2009;23(6):694–707. 10.1101/gad.1765109 19299559 PMC2661613

[39] Satheesha S, Manzella G, Bovay A, Casanova EA, Bode PK, Belle R, et al Targeting hedgehog signaling reduces self-renewal in embryonal rhabdomyosarcoma. Oncogene. 2015 10.1038/onc.2015.267 . 26189795 PMC5399168

[40] Gerber AN, Wilson CW, Li YJ, Chuang PT. The hedgehog regulated oncogenes Gli1 and Gli2 block myoblast differentiation by inhibiting MyoD-mediated transcriptional activation. Oncogene. 2007;26(8):1122–36. 10.1038/sj.onc.1209891 16964293 PMC3325095

[41] Uhmann A, Niemann H, Lammering B, Henkel C, Hess I, Rosenberger A, et al Calcitriol inhibits hedgehog signaling and induces vitamin d receptor signaling and differentiation in the patched mouse model of embryonal rhabdomyosarcoma. Sarcoma. 2012;2012:357040 10.1155/2012/357040 22550417 PMC3329653

[42] Raimondi L, Ciarapica R, De Salvo M, Verginelli F, Gueguen M, Martini C, et al Inhibition of Notch3 signalling induces rhabdomyosarcoma cell differentiation promoting p38 phosphorylation and p21(Cip1) expression and hampers tumour cell growth in vitro and in vivo. Cell Death Differ. 2012;19(5):871–81. 10.1038/cdd.2011.171 22117196 PMC3321627

[43] De Salvo M, Raimondi L, Vella S, Adesso L, Ciarapica R, Verginelli F, et al Hyper-activation of Notch3 amplifies the proliferative potential of rhabdomyosarcoma cells. PloS one. 2014;9(5):e96238 10.1371/journal.pone.0096238 24797362 PMC4010457

[44] Boyd AW, Bartlett PF, Lackmann M. Therapeutic targeting of EPH receptors and their ligands. Nat Rev Drug Discov. 2014;13(1):39–62. 10.1038/nrd4175 . 24378802

[45] Pasquale EB. Eph receptors and ephrins in cancer: bidirectional signalling and beyond. Nat Rev Cancer. 2010;10(3):165–80. 10.1038/nrc2806 20179713 PMC2921274

[46] Chiappalupi S, Riuzzi F, Fulle S, Donato R, Sorci G. Defective RAGE activity in embryonal rhabdomyosarcoma cells results in high PAX7 levels that sustain migration and invasiveness. Carcinogenesis. 2014;35(10):2382–92. 10.1093/carcin/bgu176 . 25123133

[47] Clifford N, Smith LM, Powell J, Gattenlohner S, Marx A, O'Connor R. The EphA3 receptor is expressed in a subset of rhabdomyosarcoma cell lines and suppresses cell adhesion and migration. J Cell Biochem. 2008;105(5):1250–9. 10.1002/jcb.21926 . 18814179

[48] Aslam MI, Abraham J, Mansoor A, Druker BJ, Tyner JW, Keller C. PDGFRbeta reverses EphB4 signaling in alveolar rhabdomyosarcoma. Proceedings of the National Academy of Sciences of the United States of America. 2014;111(17):6383–8. 10.1073/pnas.1403608111 24733895 PMC4035936

[49] Berardi AC, Marsilio S, Rofani C, Salvucci O, Altavista P, Perla FM, et al Up-regulation of EphB and ephrin-B expression in rhabdomyosarcoma. Anticancer Res. 2008;28(2A):763–9. PubMed PMID: . 18507018

[50] Lopez-Atalaya JP, Ito S, Valor LM, Benito E, Barco A. Genomic targets, and histone acetylation and gene expression profiling of neural HDAC inhibition. Nucleic acids research. 2013;41(17):8072–84. 10.1093/nar/gkt590 23821663 PMC3783173

[51] Wang Z, Zang C, Cui K, Schones DE, Barski A, Peng W, et al Genome-wide mapping of HATs and HDACs reveals distinct functions in active and inactive genes. Cell. 2009;138(5):1019–31. 10.1016/j.cell.2009.06.049 19698979 PMC2750862

[52] Hedrick E, Crose L, Linardic CM, Safe S. Histone Deacetylase Inhibitors Inhibit Rhabdomyosarcoma by Reactive Oxygen Species-Dependent Targeting of Specificity Protein Transcription Factors. Mol Cancer Ther. 2015;14(9):2143–53. 10.1158/1535-7163.MCT-15-0148 . 26162688 PMC4618474

[53] Chen EY, Dobrinski KP, Brown KH, Clagg R, Edelman E, Ignatius MS, et al Cross-species array comparative genomic hybridization identifies novel oncogenic events in zebrafish and human embryonal rhabdomyosarcoma. PLoS genetics. 2013;9(8):e1003727 10.1371/journal.pgen.1003727 24009521 PMC3757044

[54] Mizgireuv IV, Revskoy SY. Transplantable tumor lines generated in clonal zebrafish. Cancer Res. 2006;66(6):3120–5. 10.1158/0008-5472.CAN-05-3800 . 16540662

[55] Smyth GK. Linear models and empirical bayes methods for assessing differential expression in microarray experiments. Stat Appl Genet Mol Biol. 2004;3:Article3. 10.2202/1544-6115.1027 . 16646809

[56] Carvalho BS, Irizarry RA. A framework for oligonucleotide microarray preprocessing. Bioinformatics. 2010;26(19):2363–7. 10.1093/bioinformatics/btq431 20688976 PMC2944196

[57] Ritchie ME, Diyagama D, Neilson J, van Laar R, Dobrovic A, Holloway A, et al Empirical array quality weights in the analysis of microarray data. BMC Bioinformatics. 2006;7:261 10.1186/1471-2105-7-261 16712727 PMC1564422

