# Supplementary figures and images for: Histone Deacetylase Inhibitors Antagonize Distinct Pathways to Suppress Tumorigenesis of Embryonal Rhabdomyosarcoma
Source: PLoS One. 2015 Dec 4;10(12):e0144320. doi: 10.1371/journal.pone.0144320 (PMC4670218; doi:10.1371/journal.pone.0144320)

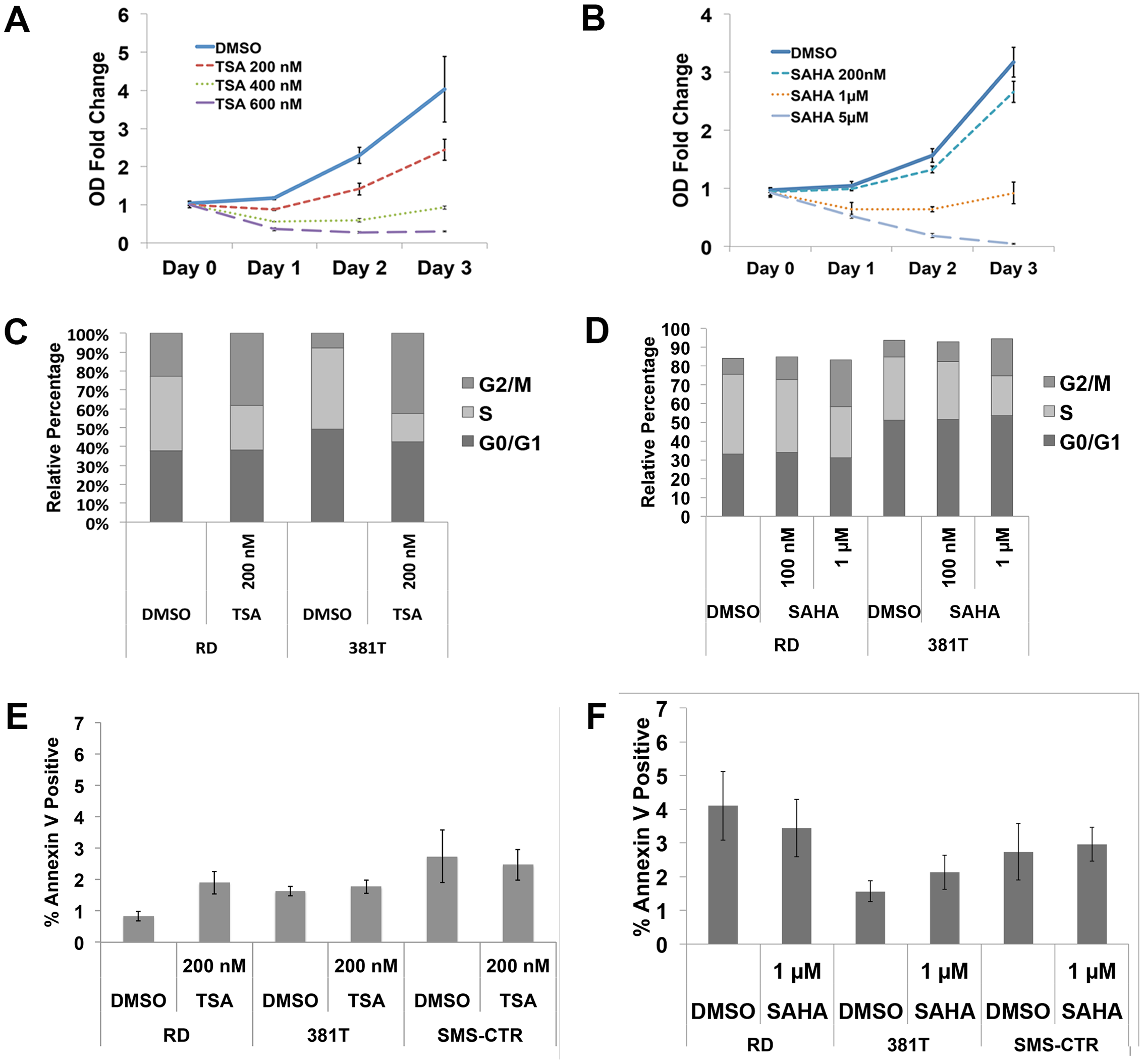

Supplement: S1 Fig — (A-B) CellTiter-Glo viability assays on RD cells treated with DMSO, 3 doses of TSA (A) or 3 doses of SAHA (B). (C-D) Cell cycle analysis of RD and 381T cells treated with TSA (C) or SAHA (D). (E-F) Annexin V analysis of RD, 381T and SMS-CTR cells treated with TSA (E) or SAHA (F). Each error bar indicates standard deviation of technical triplicates. (TIF) [file pone.0144320.s001.tif]

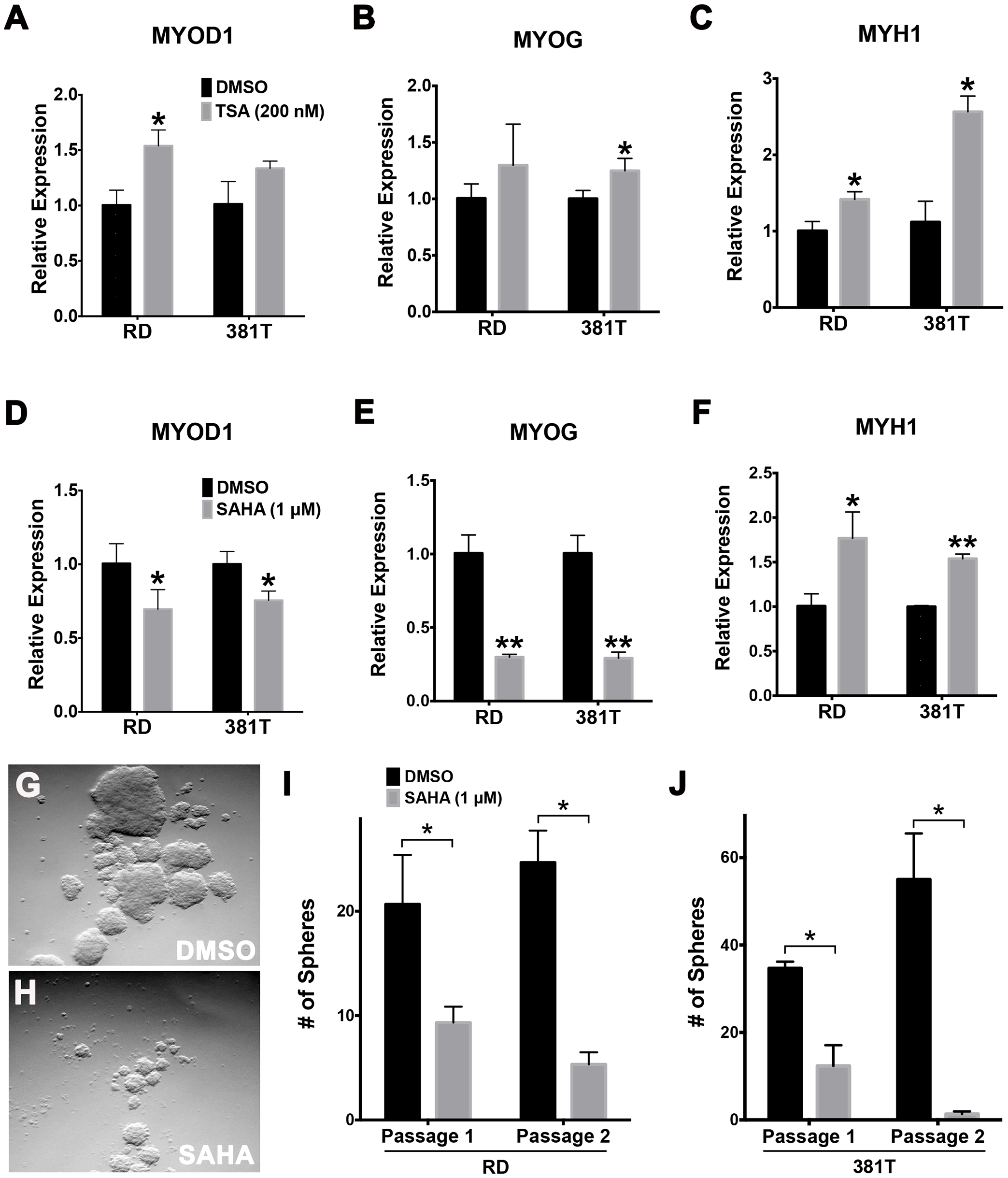

Supplement: S2 Fig — (A-F) Quantitative RT-PCR of myogenic genes MYOD1, MYOG, and MYH1 after 24 hour treatment with either DMSO or 200 nM TSA (A-C) or 1 μM SAHA (D-F). (G-H) Representative bright field images from sphere assay of ERMS cells treated with DMSO or 1 μM SAHA. (I-J) Serial replating of RD and 381T spheres treated with 1 μM SAHA. Each error bar denotes standard deviation of experimental triplicate. * indicates p < 0.05. ** indicates p < 0.01. (TIF) [file pone.0144320.s002.tif]

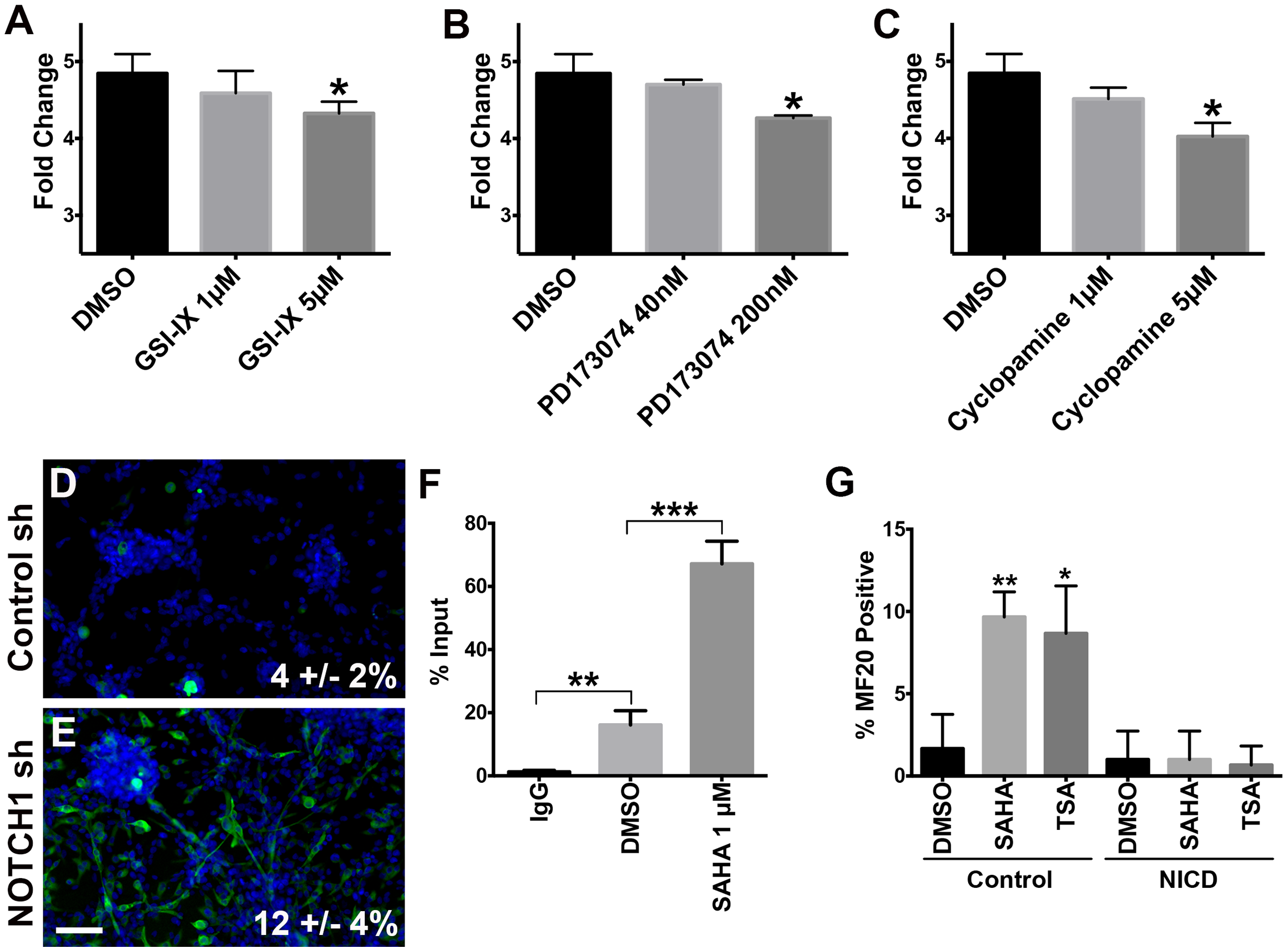

Supplement: S3 Fig — (A-C) Summary of CellTiter-Glo viability assays of RD cells treated with DMSO or GSI-IX (A), PD173074 (B), or cyclopamine (C). Fold change in ATP luminescence signal intensity over 4 days is shown. Error bars indicate standard deviation of technical triplicates. (D-E) Representative images of MF20 immuofluorescence of RD cells harboring control shRNA (D) and NOTCH1 shRNA (E). Quantitation of percent MF20+ cells including standard deviation is shown on each panel. Scale bar indicates 20 μm. (F) ChIP assay showing differential binding of acetyl-histone H3 (Lys9) on NOTCH1 promoter in RD cells treated with DMSO or 1 μM SAHA. Rabbit IgG was used as a negative control for chromatin immunoprecipitation. (G) Summary of MF20 IF of control GFP-overexpressing and NICD-overexpressing 381T cells treated with DMSO, 200 nM TSA or 1 μM SAHA. Error bar in each panel indicates standard deviation of experimental triplicates. * indicates p < 0.05. ** indicates p < 0.01. *** indicates p < 0.001. (TIF) [file pone.0144320.s003.tif]

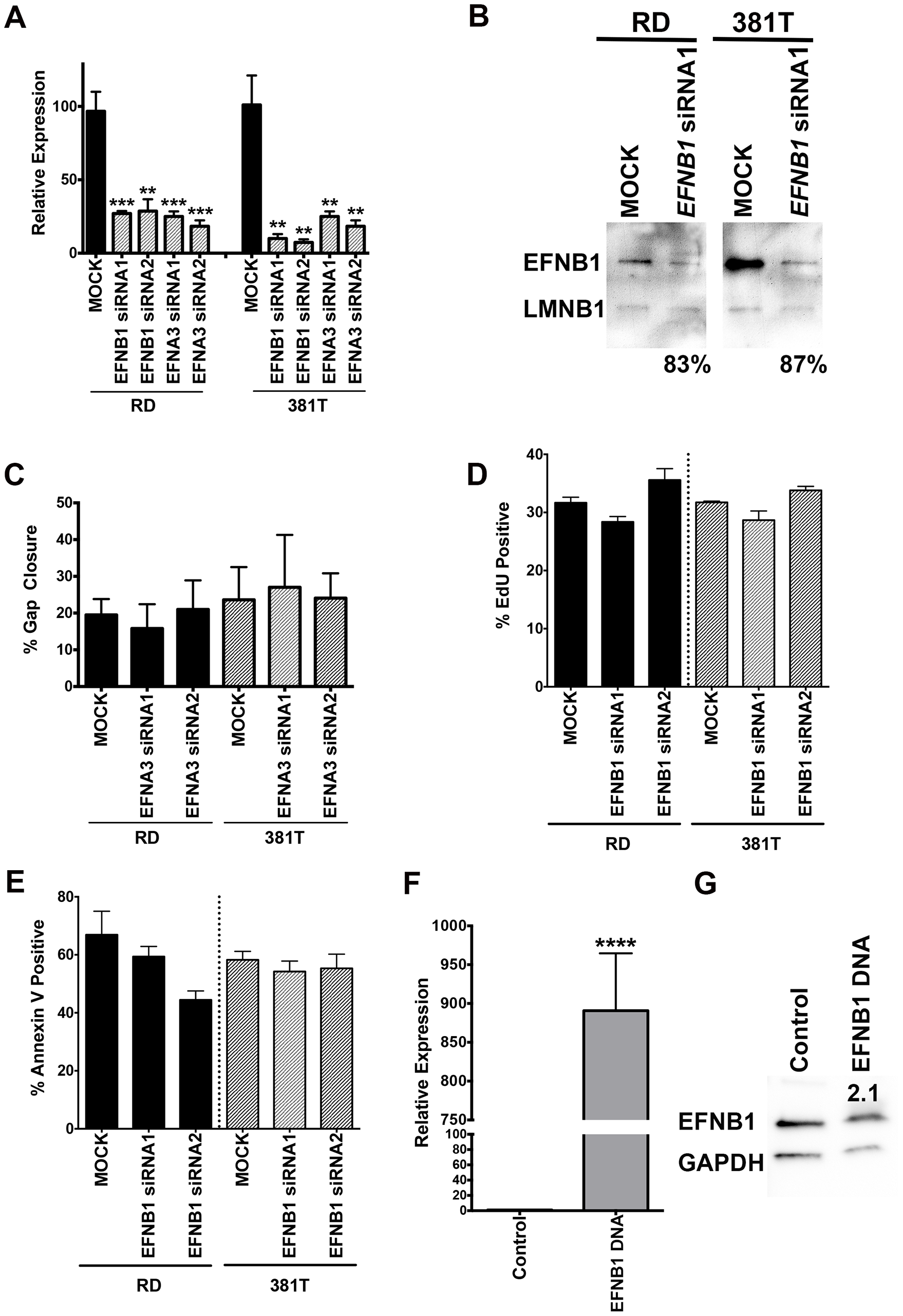

Supplement: S4 Fig — (A) Quantitative RT-PCR showing effective knockdown of EFNB1 and EFNA3 mRNA expression using 2 independent gene-specific siRNAs. Levels are shown in comparison to mock-treated samples. (B) Western blot analysis showing effective knockdown of EFNB1 protein level in RD and 381T cells by EFNB1 siRNA. Each band intensity was normalized to Lamin B1 (LMNB1) loading control. % knockdown of EFNB1 relative to mock treatment is indicated. (C) Summary of scratch assay performed on RD and 381T cells with EFNA3 knockdown by 2 independent siRNAs. (D) EdU flow cytometry-based assay to assess proliferation rate of RD and 381T cells with EFNB1 knockdown by 2 independent siRNAs. (E) Annexin V flow cytometry-based assay to assess the extent of apoptosis in RD and 381T cells with EFNB1 knockdown by 2 independent siRNAs. (F) Quantitative RT-PCR confirming increased expression of EFNB1 mRNA in the overexpression cell line. (G) Western blot analysis confirming increased expression of EFNB1 protein in the overexpression cell line. Each band intensity was normalized to GAPDH loading control. Fold expression compared to control GFP-overexpressing cell line is indicated. Error bar in each panel indicates standard deviation of experimental triplicates. ** indicates p < 0.01. *** indicates p < 0.001. **** indicates p < 0.0001. (TIF) [file pone.0144320.s004.tif]

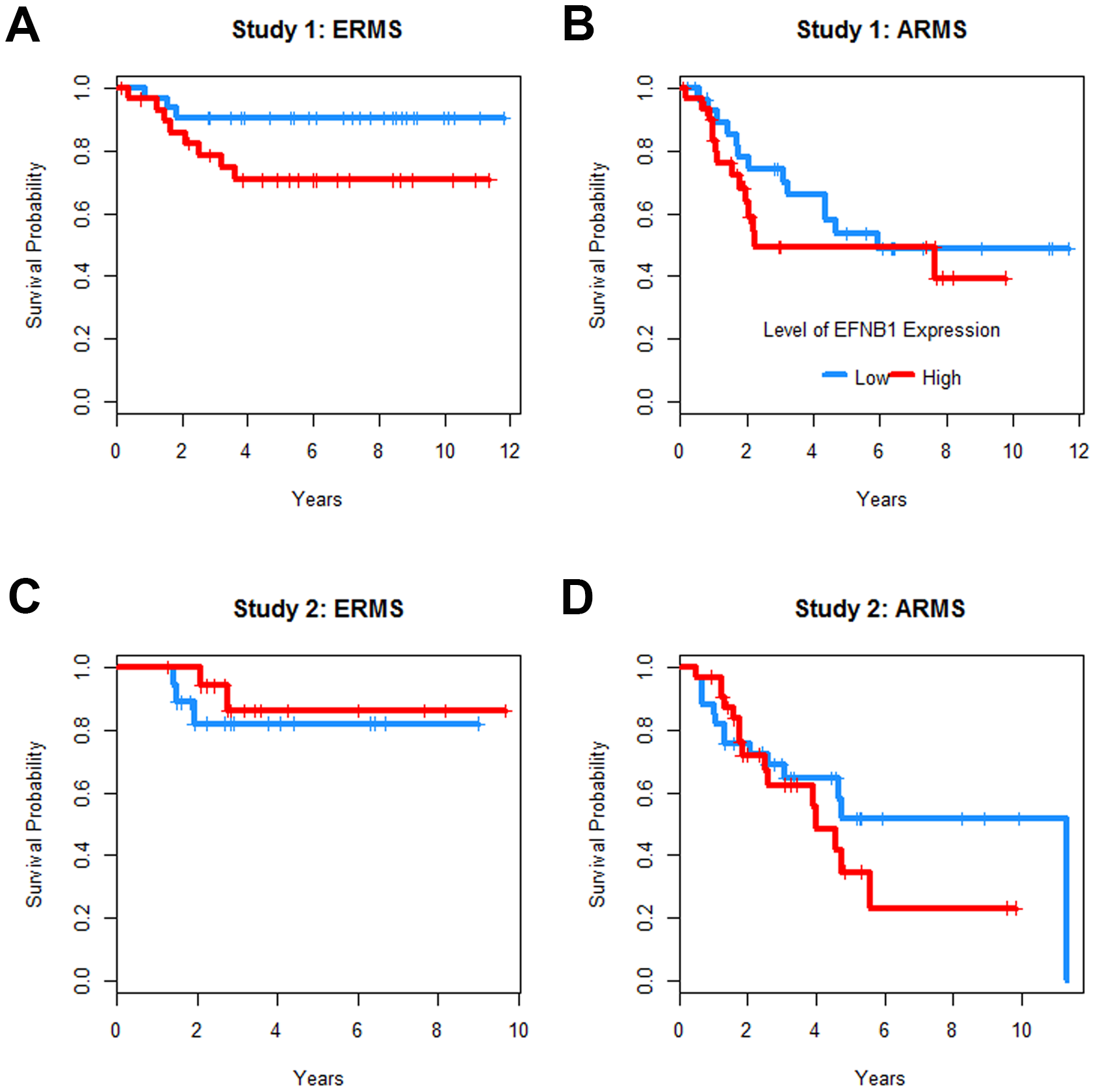

Supplement: S5 Fig — Kaplan-Meier curves comparing the probability of survival between levels of EFNB1 expression within ERMS and ARMS patients (A-B) Results for the Davicioni study: ERMS (n = 62, 11 deaths) and ARMS (n = 62, 27 deaths). (C-D) Results for the Williamson study: ERMS (n = 36, 5 deaths) and ARMS (n = 65, 29 deaths). Red: high EFNB1 expression. Blue: low EFNB1 expression. (TIF) [file pone.0144320.s005.tif]
