# Supplementary material for: Histone Deacetylase Inhibitors Antagonize Distinct Pathways to Suppress Tumorigenesis of Embryonal Rhabdomyosarcoma
Source: PLoS One. 2015 Dec 4;10(12):e0144320. doi: 10.1371/journal.pone.0144320 (PMC4670218; doi:10.1371/journal.pone.0144320)
Supplement: S1 Table — (PDF) [file pone.0144320.s006.pdf]

Supplemental Table 1: Primers used in quantitative PCR

Species	Gene Symbol	Official Full Name	Amplification Site	Fwd Sequence	Rev Sequence
Homo sapiens	*CPA4* (ID: 51200)	carboxypeptidase A4	Coding region	CAAATTTGATGCCGTTGTCA	AAGCTCTGGCTTCTGTGTCG
	DNER (ID: 92737)	delta/notch-like EGF repeat containing	Coding region	AGTTGCCATGGTGACAAGG	CTACAGCTGCACCTGCCC
	EFNA3 (ID: 1944)	ephrin-A3	Coding region	ACCCCCGAGCTGTTGTAGTG	GCATGCGGTGTACTGGAAC
	EFNB1 (ID: 1947)	ephrin-B1	Promoter	GCACCTTTGGTTCCTCTCTG	CCCTCCCCATCTACACTGAA
			Coding region	GCAGATGATGTCCAGCTTGT	AAGAACCTGGAGCCCGTATC
	FGFR1 (ID: 2260)	fibroblast growth factor receptor 1	Coding region	GGAAGGACTCCACTTCCACA	GTCACAGCCACACTCTGCAC
	GAPDH (ID: 2597)	glyceraldehyde-3-phosphate dehydrogenase	Coding region	GGTGGTCTCCTCTGACTTCAACA	GTTGCTGTAGCCAAATTCGTTGT
	HES1 (ID: 3280)	hes family bHLH transcription factor 1	Coding region	ACGTGCGAGGGCGTTAATAC	GGGGTAGGTCATGGCATTGA
	HEY1 (ID: 23462)	hes-related family bHLH transcription factor with YRPW motif 1	Coding region	ATCTGCTAAGCTAGAAAAAGCCG	GTGCGCGTCAAAGTAACCT
	HEY2 (ID: 23493)	hes-related family bHLH transcription factor with YRPW motif 2	Coding region	AAGGCGTCGGGATCGGATAA	AGAGCGTGTGCGTCAAAGTAG
	IGF2BP1 (ID: 10642)	insulin-like growth factor 2 mRNA bindi	Coding region	CAAAGGAGCCGGAAAATTCAAAT	CGTCTCACTCTCGGTGTTCA
	MPP4 (ID: 58538)	membrane protein, palmitoylated 4 (MAGUK p55 subfamily member 4)	Coding region	TAGGGTTGACTTGAGGCAGG	CCCGAAAAATCTCAGACCCT
	MYH1 (ID:4619)	myosin, heavy chain 1, skeletal muscle, adult	Coding region	GTGAGAGGCTGGAAGAAGCCGTG	TCCTCCAGGTCCCTGCGCATT
	MYH4 (ID: 4622)	myosin, heavy chain 4, skeletal muscle	Promoter	GGTCGAGAGTTCGAGACCAG	CAGGGTGTCTCCTTCAAAGC
	MYOD1 (ID: 4654)	myogenic differentiation 1	Promoter	GACTGGCGGAATATCAGAGC	GAGCGGCTGTAGAAATCAGG
			Coding region	AGCACTACAGCGGCGACT	GCGACTCAGAAGGCACGTC
	MYOG (ID: 4656)	myogenin (myogenic factor 4)	Promoter	CACGGAGGACAACTGGCTAT	ACAGGGCTCCTGGTATTGTG
			Coding region	CCTGCCGTGGGCGTGTAAGG	GGACTGCAGGAGGCGCTGTG
	NOTCH1 (ID:4851)	notch 1	Promoter	TGTTCCGCTCACACAAAGAG	GATACAGGATGCCTGGGAGA
	PTPRN (ID: 5798)	protein tyrosine phosphatase, receptor type, N	Coding region	GCTCCATCTCCTGAGAGATCA	CAAGTCACCTCCCCAGTTCT
	SEMA3C (ID: 10512)	sema domain, immunoglobulin domain (Ig), short basic domain, secreted, (semaphorin) 3C	Coding region	ATCCGGTCCTGATCTTCATC	CAGCCCCAAGCAAGAGTTTA
	SMO (ID: 6608)	smoothened, frizzled class receptor	Coding region	GTCATTCTCACACTTGGGCA	AAGCTCGTGCTCTGGTCG
	TAZ (ID: 6901)	tafazzin	Coding region	ATTCATCGCCTTCCTAGGGT	GGCTGGGAGATGACCTTCAC
	TNFRSF12A (ID: 51330)	tumor necrosis factor receptor superfamily, member 12A	Coding region	CAAGACGCGCAGTCCAT	CTCGTGCTGGGGCTCTG
Danio rerio	*18S rRNA*	18S rRNA	Coding region	TCGCTAGTTGGCATCGTTTATG	CGGAGGTTCGAAGACGATCA
	myod1 (ID: 30513)	myogenic differentiation 1	Coding region	CAGTGGAGACTCTGATGCTTCCAG	AGCTGTCATAGCTGTTCCGTCTTC
	myog (ID: 30200)	myogenin	Coding region	GTGGACAGCATAACGGGAACAG	TCTGAAGGTAACGGTGAGTCGG
